# Childhood gene-environment interactions and age-dependent effects of genetic variants associated with refractive error and myopia: The CREAM Consortium

**DOI:** 10.1038/srep25853

**Published:** 2016-05-13

**Authors:** Qiao Fan, Xiaobo Guo, J. Willem L. Tideman, Katie M. Williams, Seyhan Yazar, S. Mohsen Hosseini, Laura D. Howe, Beaté St Pourcain, David M. Evans, Nicholas J. Timpson, George McMahon, Pirro G. Hysi, Eva Krapohl, Ya Xing Wang, Jost B. Jonas, Paul Nigel Baird, Jie Jin Wang, Ching-Yu Cheng, Yik-Ying Teo, Tien-Yin Wong, Xiaohu Ding, Robert Wojciechowski, Terri L. Young, Olavi Pärssinen, Konrad Oexle, Norbert Pfeiffer, Joan E. Bailey-Wilson, Andrew D. Paterson, Caroline C. W. Klaver, Robert Plomin, Christopher J. Hammond, David A. Mackey, Mingguang He, Seang-Mei Saw, Cathy Williams, Jeremy A. Guggenheim, The CREAM Consortium, Akira Meguro, Alan F. Wright, Alex W. Hewitt, Alvin L. Young, Amutha Barathi Veluchamy, Andres Metspalu, Andrew D. Paterson, Angela Döring, Anthony P. Khawaja, Barbara E. Klein, Beate St Pourcain, Brian Fleck, Caroline C. W. Klaver, Caroline Hayward, Cathy Williams, Cécile Delcourt, Chi Pui Pang, Chiea-Chuen Khor, Ching-Yu Cheng, Christian Gieger, Christopher J. Hammond, Claire L. Simpson, Cornelia M. van Duijn, David A. Mackey, David M. Evans, Dwight Stambolian, Emily Chew, E-Shyong Tai, Eva Krapohl, Evelin Mihailov, George Davey Smith, George McMahon, Ginevra Biino, Harry Campbell, Igor Rudan, Ilkka Seppälä, Jaakko Kaprio, James F. Wilson, Jamie E. Craig, J. Willem L. Tideman, Janina S. Ried, Jean-François Korobelnik, Jeremy A. Guggenheim, Jeremy R. Fondran, Jie Jin Wang, Jiemin Liao, Jing Hua Zhao, Jing Xie, Joan E. Bailey-Wilson, John P. Kemp, Jonathan H. Lass, Jost B. Jonas, Jugnoo S. Rahi, Juho Wedenoja, Kari-Matti Mäkelä, Kathryn P. Burdon, Katie M Williams, Kay-Tee Khaw, Kenji Yamashiro, Konrad Oexle, Laura D. Howe, Li Jia Chen, Liang Xu, Lindsay Farrer, M. Kamran Ikram, Margaret M. Deangelis, Margaux Morrison, Maria Schache, Mario Pirastu, Masahiro Miyake, Maurice K. H. Yap, Maurizio Fossarello, Mika Kähönen, Milly S. Tedja, Mingguang He, Nagahisa Yoshimura, Nicholas G. Martin, Nicholas J. Timpson, Nick J. Wareham, Nobuhisa Mizuki, Norbert Pfeiffer, Olavi Pärssinen, Olli Raitakari, Ozren Polasek, Pancy O. Tam, Paul J. Foster, Paul Mitchell, Paul Nigel Baird, Peng Chen, Pirro G. Hysi, Phillippa Cumberland, Puya Gharahkhani, Qiao Fan, René Höhn, Rhys D. Fogarty, Robert N. Luben, Robert P. Igo Jr, Robert Plomin, Robert Wojciechowski, Ronald Klein, S. Mohsen Hosseini, Sarayut Janmahasatian, Seang-Mei Saw, Seyhan Yazar, Shea Ping Yip, Sheng Feng, Simona Vaccargiu, Songhomitra Panda-Jonas, Stuart MacGregor, Sudha K. Iyengar, Taina Rantanen, Terho Lehtimäki, Terri L. Young, Thomas Meitinger, Tien-Yin Wong, Tin Aung, Toomas Haller, Veronique Vitart, Vinay Nangia, Virginie J. M. Verhoeven, Vishal Jhanji, Wanting Zhao, Wei Chen, Xiangtian Zhou, Xiaobo Guo, Xiaohu Ding, Ya Xing Wang, Yi Lu, Yik-Ying Teo, Zoran Vatavuk

**Affiliations:** 1 Centre for Quantitative Medicine, Duke-NUS Medial School, Singapore; 2 Department of Statistical Science, School of Mathematics & Computational Science, Sun Yat-Sen University, Guangzhou, China; 3 State Key Laboratory of Ophthalmology, Zhongshan Ophthalmic Center, Sun Yat-sen University, Guangzhou, China; 4 Department of Epidemiology, Erasmus Medical Center, Rotterdam, The Netherlands; 5 Department of Ophthalmology, Erasmus Medical Center, Rotterdam, The Netherlands; 6 Department of Ophthalmology, King’s College London, St Thomas’ Hospital campus, London, UK; 7 Department of Twin Research and Genetic Epidemiology, King’s College London School of Medicine, London, UK; 8 Centre for Ophthalmology and Visual Science, Lions Eye Institute, University of Western Australia, Perth, Australia; 9 Genetics and Genome Biology Program, Hospital for Sick Children and University of Toronto, Toronto, Ontario, Canada; 10 MRC Integrative Epidemiology Unit (IEU) at the University of Bristol, Bristol, UK; 11 School of Social and Community Medicine, University of Bristol, Bristol, UK; 12 Max Planck Institute for Psycholinguistics, Wundtlaan 1, 6525 XD Nijmegen, The Netherlands; 13 University of Queensland Diamantina Institute, Translational Research Institute, Brisbane, Queensland, Australia; 14 MRC Social, Genetic and Developmental Psychiatry Centre, Institute of Psychiatry, Psychology & Neuroscience, King’s College London, London, UK; 15 Beijing Institute of Ophthalmology, Beijing Tongren Hospital, Capital Medical University, Beijing, China; 16 Beijing Ophthalmology and Visual Science Key Lab, Beijing, China; 17 Department of Ophthalmology, Medical Faculty Mannheim, Ruprecht-Karls-University Heidelberg, Mannheim, Germany; 18 Centre for Eye Research Australia (CERA), University of Melbourne, Royal Victorian Eye and Ear Hospital, Melbourne, Victoria, Australia; 19 Centre for Vision Research, Department of Ophthalmology and Westmead Institute for Medical Research, University of Sydney, Sydney, Australia; 20 Department of Ophthalmology, National University Health Systems, National University of Singapore, Singapore; 21 Singapore Eye Research Institute, Singapore National Eye Centre, Singapore, Singapore; 22 Department of Statistics and Applied Probability, National University of Singapore, Singapore, Singapore; 23 Saw Swee Hock School of Public Health, National University Health Systems, National University of Singapore, Singapore, Singapore; 24 Computational and Statistical Genomics Branch, National Human Genome Research Institute, National Institutes of Health, Baltimore, MD, USA; 25 Department of Epidemiology, Johns Hopkins Bloomberg School of Public Health, Baltimore, Maryland, USA; 26 Wilmer Eye Institute, Johns Hopkins School of Medicine, Baltimore, MD; 27 Ophthalmology and Visual Sciences, University of Wisconsin-Madison, Madison, WI, USA; 28 Duke-National University of Singapore Graduate Medical School, Singapore, Singapore; 29 Department of Health Sciences and Gerontology Research Center, University of Jyväskylä, Jyväskylä, Finland; 30 Department of Ophthalmology, Central Hospital of Central Finland, Jyväskylä, Finland; 31 Institute of Human Genetics, Technical University Munich, Munich, Germany; 32 Department of Ophthalmology, University Medical Center Mainz, Mainz, Germany; 33 School of Optometry & Vision Sciences, Cardiff University, Cardiff, UK; 34 Department of Ophthalmology, Yokohama City University School of Medicine, Yokohama, Kanagawa, Japan.; 35 Medical Research Council Human Genetics Unit, Institute of Genetics and Molecular Medicine, University of Edinburgh, Edinburgh, UK.; 36 Department of Ophthalmology and Visual Sciences, The Chinese University of Hong Kong Hong Kong Eye Hospital, Kowloon, Hong Kong.; 37 Estonian Genome Center, University of Tartu, Tartu, Estonia.; 38 Institute of Genetic Epidemiology, Helmholtz Zentrum München—German Research Center for Environmental Health, Neuherberg, Germany.; 39 Department of Public Health and Primary Care, Institute of Public Health, University of Cambridge School of Clinical Medicine, Cambridge, UK.; 40 Princess Alexandra Eye Pavilion, Edinburgh, UK.; 41 Université de Bordeaux, Bordeaux, France.; 42 INSERM (Institut National de la Santé Et de la Recherche Médicale), ISPED (Institut de Santé Publique d’Épidémiologie et de Développement), Centre INSERM U897- Epidemiologie-Biostatistique, Bordeaux, France.; 43 Division of Human Genetics, Genome Institute of Singapore, Singapore, Singapore.; 44 Department of Ophthalmology and Visual Sciences, University of Utah, Moran Eye Center, Salt Lake City, USA.; 45 Department of Ophthalmology, University of Pennsylvania, Philadelphia, Pennsylvania, USA.; 46 Department of Genetics, Case Western Reserve University, Cleveland, Ohio, USA.; 47 Department of Medicine, National University of Singapore, Singapore, Singapore.; 48 Institute of Molecular Genetics, National Research Council, Pavia, Italy.; 49 Centre for Global Health Research, The Usher Institute for Population Health Sciences and Informatics, University of Edinburgh, Scotland, UK.; 50 Department of Clinical Chemistry, Fimlab laboratories and School of Medicine, University of Tampere, Tampere, Finland.; 51 Department of Public Health, University of Helsinki, Helsinki, Finland.; 52 Department of Ophthalmology, Flinders University, Adelaide, Australia.; 53 Department of Epidemiology and Biostatistics, Case Western Reserve University, Cleveland, Ohio, USA.; 54 MRC Epidemiology Unit, Institute of Metabolic Sciences, University of Cambridge, Cambridge, UK.; 55 Department of Ophthalmology and Visual Sciences, Case Western Reserve University and University Hospitals Eye Institute, Cleveland, Ohio, USA.; 56 Institute of Child Health, University College London, London, UK.; 57 Institute of Ophthalmology, Moorfields Eye Hospital, London, UK.; 58 Ulverscroft Vision Research Group, University College London, London, UK.; 59 Department of Ophthalmology, Helsinki University Central Hospital, Helsinki, Finland.; 60 Department of Ophthalmology and Visual Sciences, Kyoto University Graduate School of Medicine, Kyoto, Japan.; 61 Department of Ophthalmology and Visual Sciences, John Moran Eye Center, University of Utah, Salt Lake City, Utah, USA.; 62 Institute of Genetic and Biomedic Research, National Research Council, Cagliari, Italy.; 63 Centre for Myopia Research, School of Optometry, The Hong Kong Polytechnic University, Hong Kong, Hong Kong.; 64 University Hospital ‘San Giovanni di Dio’, Cagliari, Italy.; 65 Department of Clinical Physiology, Tampere University Hospital and School of Medicine, University of Tampere, Tampere, Finland.; 66 Genetic Epidemiology Laboratory, QIMR Berghofer Medical Research Institute, Herston, Brisbane, Queensland, Australia.; 67 Research Centre of Applied and Preventive Medicine, University of Turku, Turku, Finland.; 68 Department of Clinical Physiology and Nuclear Medicine, Turku University Hospital, Turku, Finland.; 69 Faculty of Medicine, University of Split, Split, Croatia.; 70 Division of Genetics and Epidemiology, UCL Institute of Ophthalmology, London, UK.; 71 NIHR Biomedical Research Centre, Moorfields Eye Hospital NHS Foundation Trust and UCL Institute of Ophthalmology, London, UK.; 72 Statistical Genetics Laboratory, QIMR Berghofer Medical Research Institute, Herston, Brisbane, Queensland, Australia.; 73 Department of Health Technology and Informatics, The Hong Kong Polytechnic University, Hong Kong, Hong Kong.; 74 Department of Pediatric Ophthalmology, Duke Eye Center For Human Genetics, Durham, North Carolina, USA.; 75 Suraj Eye Institute, Nagpur, Maharashtra, India.; 76 Gerontology Research Center, University of Jyväskylä, Jyväskylä, Finland.; 77 Department of Radiology, Southwest Hospital, The Third Military Medical University, Chongqing, China.; 78 Department of Ophthalmology, Sisters of Mercy University Hospital, Zagreb, roatia.

## Abstract

Myopia, currently at epidemic levels in East Asia, is a leading cause of untreatable visual impairment. Genome-wide association studies (GWAS) in adults have identified 39 loci associated with refractive error and myopia. Here, the age-of-onset of association between genetic variants at these 39 loci and refractive error was investigated in 5200 children assessed longitudinally across ages 7–15 years, along with gene-environment interactions involving the major environmental risk-factors, nearwork and time outdoors. Specific variants could be categorized as showing evidence of: (a) early-onset effects remaining stable through childhood, (b) early-onset effects that progressed further with increasing age, or (c) onset later in childhood (N = 10, 5 and 11 variants, respectively). A genetic risk score (GRS) for all 39 variants explained 0.6% (P = 6.6E–08) and 2.3% (P = 6.9E–21) of the variance in refractive error at ages 7 and 15, respectively, supporting increased effects from these genetic variants at older ages. Replication in multi-ancestry samples (combined N = 5599) yielded evidence of childhood onset for 6 of 12 variants present in both Asians and Europeans. There was no indication that variant or GRS effects altered depending on time outdoors, however 5 variants showed nominal evidence of interactions with nearwork (top variant, rs7829127 in *ZMAT4*; P = 6.3E–04).

The refractive errors myopia and hyperopia are common visual disorders that typically require correction with spectacles, contact lenses, or refractive eye surgery. Myopia – particularly with increasing severity – is a leading cause of irreversible visual impairment and blindness due primarily to stretching and thinning of the ocular tissues within the posterior segment of the eye. These changes are associated with an increased risk of retinal detachment, chorioretinal atrophy, choroidal neovascularisation, myopic maculopathy, glaucoma and cataract^1^,^2^. Myopia is rare in infancy, usually developing during school age or in early adulthood^3^. For current generations of young adults, approximately 30–40% of individuals in Western countries^4^,^5^ and 80% of those in urban areas of East Asia^6^,^7^ have myopia.

Genome-wide association studies (GWAS) in primarily population-based samples^8^,^9^,^10^,^11^,^12^,^13^,^14^ and next-generation sequencing (NGS) studies of carefully selected high myopia pedigrees harbouring extremely rare, high penetrance disease-causing mutations^15^,^16^,^17^,^18^,^19^,^20^ have improved our understanding of the genetics of refractive error and myopia. To date at least 39 distinct loci harbouring common genetic variants showing genome-wide significant association with refractive error have been identified through GWAS. For the genetic variants that contribute most to the burden of myopia in the general population (i.e. the GWAS-identified variants) it is not yet known whether the variants act during very early life, childhood, or in adulthood. This is an important question given that knowledge of the time and mode of action of the causal variants at the associated loci is necessary for detecting children at-risk of myopia (who would benefit most from treatment intervention), and would aid the design of new therapies capable of halting myopia progression.

For environmental risk factors to which most children are exposed, inter-individual differences in genetic susceptibility may account for some of the phenotypic variance^21^. Exposure to nearwork, i.e. reading and other tasks requiring prolonged near vision, has long been proposed as an environmental risk factor for myopia to which children are ubiquitously exposed during their schooling. The total duration of reading, the period of continuous reading, the reading distance between the text and the eyes, and variation in nearwork exposure outside of the school day have each been shown to be associated with refractive error or myopia progression^22^,^23^. The other most strongly implicated environmental risk factor for myopia is insufficient time spent outdoors^24^,^25^,^26^, and it has been suggested that time spent outdoors and time spent performing nearwork activities together underlie the robust association between myopia and educational achievement^2^,^27^. Gene-environment (GxE) interactions – which in this project we define as marker-phenotype associations whose effects differ statistically depending on whether individuals have been exposed to a high vs. low level of an environmental risk factor – may contribute extensively to variation in disease susceptibility^28^. Given the recent identification of gene-environment interactions involving nearwork or level of education, a key question in myopia research currently is whether GxE interactions contribute to the rising prevalence of myopia and to the higher incidence rate observed in young Asian populations as compared to their European counterparts.

We carried out analyses of pediatric/adolescent cohorts collaborating in the Consortium for Refractive Error And Myopia (CREAM) to investigate whether the top index variants at the 39 loci previously identified in GWAS meta-analyses of adults have early-onset effects manifest during childhood. We also tested for evidence of GxE interactions involving either nearwork or time spent outdoors. A single large cohort with longitudinal measurements of refractive error over much of childhood was used for the primary analyses. Meta-analyses of cross-sectional samples were then used to test for replication.

## Methods

### Participants and phenotypes

All participants were aged <25 years-old and none had been included in the earlier CREAM meta-analysis of refractive error^9^, which only included individuals >25 years of age. Details of the participant recruitment and phenotypic assessment are presented in the Supplementary Information. The study was conducted in accordance with the Declaration of Helsinki, and all participants provided informed consent. The experimental protocols for the study were approved by the respective ethical review boards at host institutions, as follows. ALSPAC, the ALSPAC Ethics and Law Committee and the Local Research Ethics Committees; BATS, the Human Research Ethics Committee at the QIMR Berghofer Medical Research Institute; GZT, the Ethics Review Board of the Zhongshan Ophthalmic Center of Sun Yat-Sen University; RAINE, the Human Research Ethics Committee at the University of Western Australia; SCORM and STARS, the Institutional Review Boards of the Singapore Eye Research Institute, Singapore General Hospital, National University of Singapore, and the National Healthcare Group, Singapore; TEDS, the Institute of Psychiatry ethics committee; TEST, the Royal Victorian Eye and Ear Hospital, the University of Tasmania, and the Australian Twin Registry; WESDR, the Health Sciences Institution Review Board of the University of Wisconsin, Madison.

Participants underwent cycloplegic autorefraction (RAINE, TEST, BATS, GZT, SCORM, STARS) or non-cycloplegic autorefraction (ALSPAC) or subjective refraction (TEDS, WESDR) and the spherical equivalent refractive error averaged between the two eyes was calculated. Parental questionnaires that included items on time spent engaged in nearwork outside of school, and time spent in outdoor activities were used to classify children as spending a high or low amount of time performing nearwork (Table S6) or outdoors (Table S7) each day. Classification was done within each cohort separately, using a median split (“low” group, exposure below median level; “high” group, exposure above median level).

### Genetic analysis

DNA samples obtained from blood or saliva were genotyped using either an Illumina or Affymetrix high-density single nucleotide polymorphism (SNP) array, and genotypes at untyped markers were imputed using the 1000-Genomes Project reference panel (see Table S5 for details). Stringent quality control procedures (e.g. imputation quality r^2^ or info score >0.5) were applied to each cohort separately (Supplementary Information). 39 SNPs that showed genome-wide significant association with refractive error in the general adult population in two previous GWAS analyses^8^,^9^ were selected for evaluation (Table S1).

### Cross-sectional models and meta-analyses

For each of the 8 cross-sectional cohorts separately, single SNP tests of association with refractive error were conducted using the following linear regression model:




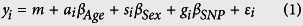




where *y*_*i*_ is the spherical equivalent refractive error of the *i*^th^ participant, of age *a*_*i*_ and sex *s*_*i*_ and with *g*_*i*_ their risk allele dosage on the scale 0–2 for the test SNP, and *ε*_*i*_ the residual. Regression coefficients are indicated as *β*_*Age*_, *β*_*Sex*_, and *β*_*SNP*_ for the model parameters age, sex and SNP genotype, respectively. Additional G x E interaction models were tested for samples with information available on environmental exposures, nearwork or time outdoors (both exposures coded: 0 = low, 1 = high). For the *i*^th^ participant, using *n*_*i*_ to denote nearwork and *t*_*i*_ for time outdoors:




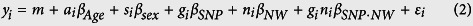







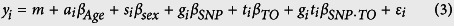




Results from the individual cohorts were meta-analyzed in 5599 individuals comprising 5 cohorts of European ancestry (BATS, RAINE, TEDS, TEST, WESDR; N = 3,143; Table 1) and 3 cohorts of Asian ancestry (GZT, SCORM, STARS; N = 2,456; Table 1) using a weighted inverse-variance, fixed effects model^29^. A random effects model was used if Cochran’s Q-test for heterogeneity yielded a P-value below 0.05.

### Longitudinal study (ALSPAC)

Refractive error was included in the clinical assessments for ages 7, 10, 11, 12 and 15 years in ALSPAC children^30^. Linear mixed models for refractive trajectory were fit as described^30^ using the *nlme* package in R^31^ for individuals (N = 5,200; Table 1) who underwent at least 3 refractive assessments and whose genotype data passed quality control filters (as described in the Supplementary Information). Briefly, SNP dosage, age and higher-order age terms (age^2^ and age^3^) were modelled as fixed effects while for each child, the difference from the average refractive error at baseline and the linear rate of change in refractive error were modelled as individual-level random effects, using an autoregressive correlation structure. To examine GxE interactions, initially, 3-way interaction models were tested that included the interaction between SNP, change-from-baseline in age, and environmental exposures (nearwork or time outdoors). If the P-value for the 3-way interaction was >0.05 then models including only 2-way interactions were tested.

Quanto^32^ was used to gauge the power to detect main and interaction effects in the ALSPAC cohort. These calculations assumed a minor allele frequency (MAF) of 0.25, a sample size of 4461 (corresponding to 5,200 minus 739 participants with missing information about time spent performing nearwork), a binary exposure affecting 39% of the cohort (equivalent to that for high vs. low nearwork exposure in ALSPAC) and a refractive error distribution with a mean of zero and a standard deviation of 1.50 D. The estimated power would be conservative given that a linear mixed model analysis will have greater power than a linear model analysis.

### Genetic risk score for all 39 SNPs

A genetic risk score was computed by summing the dosage of risk alleles for all 39 SNPs. In individuals of Asian ancestry only 31 of the 39 SNPs were polymorphic (MAF > 0.05) and therefore contributed to the genetic risk score calculation. The frequency distribution of genetic risk score in each sample was normally distributed with a mean of 36 (95% C.I. 29 to 42) alleles in Europeans and 40 (95% C.I. 37 to 42) alleles in Asians. To calculate the variance in refractive error explained by the genetic risk score at a specific age for participants in the ALSPAC cohort, refractive error at age 7.5 years (or at age 15 years) was regressed on genetic risk score using a linear model. The covariates age and sex were not associated with refractive error when included in the age 7.5 or the age 15 year model, and their inclusion did not improve the fit of either model (note that being a birth cohort, the age range was narrow). Hence these covariates were omitted. The variance explained by the genetic risk score was therefore taken as the R^2^ value for a model that included the genetic risk score as the only predictor variable.

### Pathway analysis

The genes (Table 2) implicated in having early-onset effects (N = 10 genes) or later-onset effects (N = 11 genes) in the ALSPAC discovery sample were evaluated using PANTHER Version 10.0 (release date May 15, 2015)^33^ and DAVID Version 6.7 (release date 27 Jan, 2010)^34^ to identify potential functional pathways.

## Results

### Early-onset and later-onset effects in childhood

Nine cohorts of children/adolescents were studied (Table 1). The largest of these, ALSPAC (N = 5,200), which had longitudinal data for refractive error, was used for discovery analyses, and 8 cross-sectional cohorts were used for validation. The discovery cohort had ~80% power to detect an association for a SNP with an effect size of 0.1 D and MAF of 0.25.

**Figure 1:**
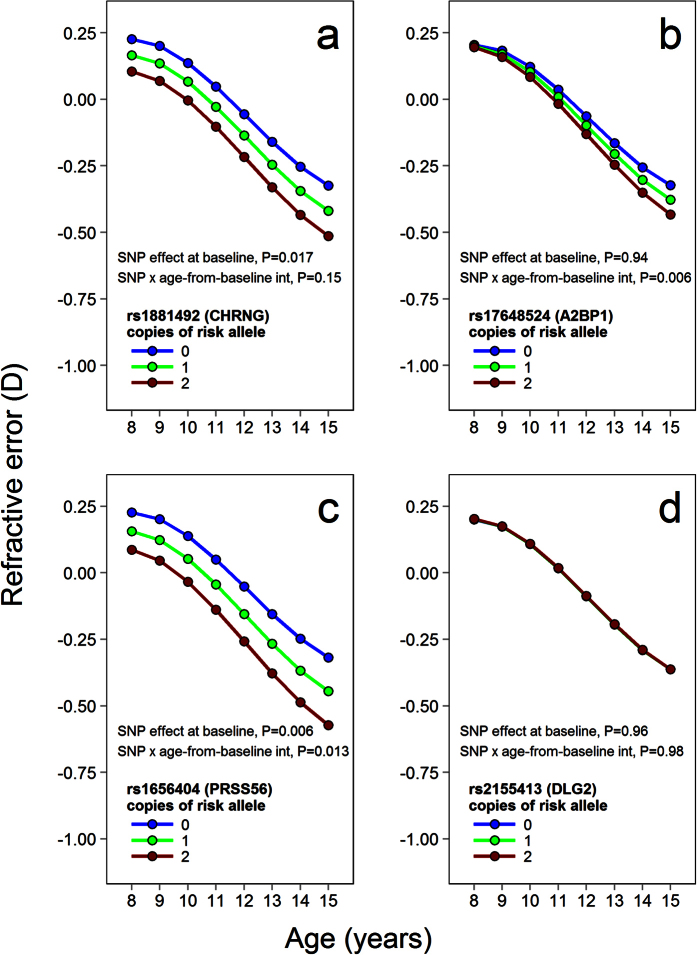
SNPs associated with early-onset and later-onset effects on refractive development during childhood. Analyses were carried out using data from longitudinal eye examinations in 5,200 ALSPAC participants. Each panel shows how refractive error trajectory varied with SNP genotype, for 4 different SNPs: rs1881492, rs17648524, rs1656404 and rs2155413. The lines in each panel show the refractive error trajectories predicted by the best-fit linear mixed model (LMM) for participants carrying the number of risk alleles indicated (0, 1 or 2). The SNPs in panels (**a**,**c**) showed an association with refractive error at baseline, i.e. evidence of early onset in childhood. The SNPs in panels (**b**,**c**) showed an age-dependent interaction with refractive error over later childhood. The SNP in panel (**d**) did not show evidence of effects during childhood.

Of the 39 SNPs examined, 16 showed evidence of onset in childhood (Table 2 and Table S2). Early-onset associations already manifest at 7.5 years of age were present for 10 SNPs (P = 4.8E–02 to P = 5.3E–03). Later-onset associations that emerged between the ages of 7.5 and 15 were noted for 11 SNPs (P = 4.9E–02 to 8.8E–04 for SNP x Age interaction). Five SNPs showed a main effect at baseline as well as later progressive effects. Examples of SNPs showing evidence of early-onset and later-onset effects are presented in Fig. 1 for early-onset *CHRNG* SNP rs1881492, later-onset *A2BP1* (also known as *RBFOX1)* rs17648524, and *PRSS56* rs1656404 with both effects. For all associated SNPs the “direction of effect” was the same as in the original GWAS^8^,^9^.

**Figure 2:**
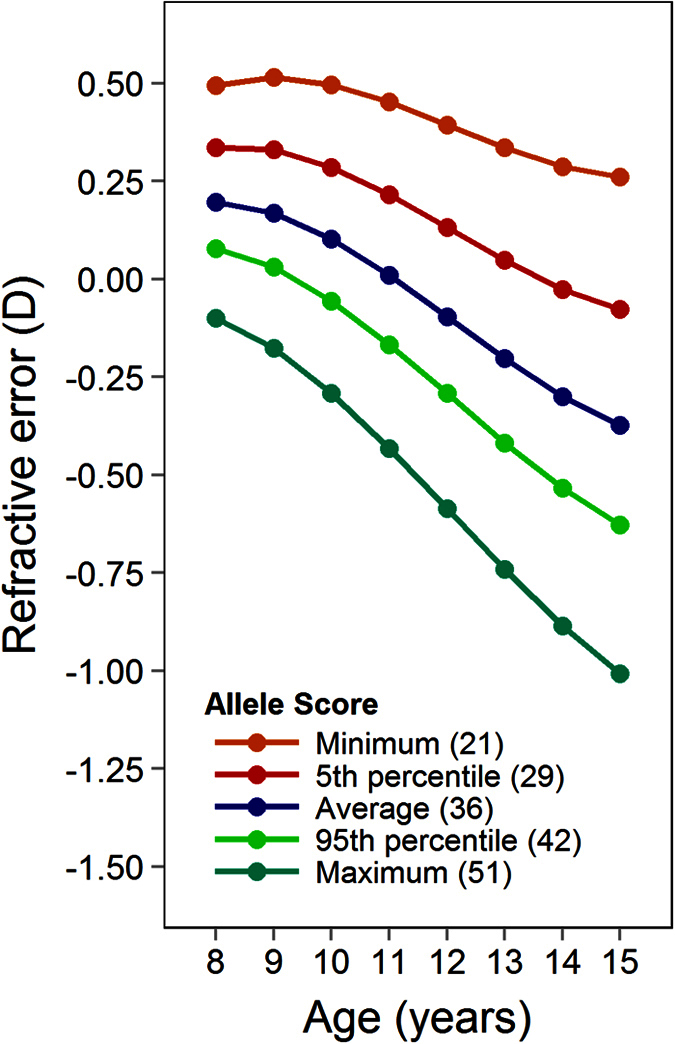
Association between a genetic risk score for 39 SNPs and refractive error trajectories in ALSPAC participants. The genetic risk score was calculated as the sum of the number of risk alleles (0–2) carried by an individual at each of the 39 myopia-susceptibility SNPs. The coloured lines show the trajectories for children carrying the number of risk alleles indicated, as predicted by the best-fit linear mixed model.

The genetic risk score was very strongly associated with refractive error both at 7.5 years of age (β = −0.018 D, 95% CI −0.012 to −0.024, P = 2.2E–9) and with increasing age (β = −0.003 D/yr, 95% CI −0.002 to −0.004, P = 5.8E–14). By the age of 15 years, the model suggested that the 39 SNPs together would produce a more than 1.0 D difference in refractive error between participants carrying the lowest and highest number of risk alleles observed (Fig. 2). At age 7.5 years the genetic risk score explained 0.6% of the variation in refractive error (N = 4,566; P = 6.6E–08); at age 15 years the corresponding figure was 2.3% (N = 3,666; P = 6.9E–21).

For validation we tested the genetic risk score and 12 of the 16 above SNPs (4 were nearly monomorphic in Asians) in the 8 multi-ethnic cross-sectional study cohorts (combined N = 5,599; Table 1). The average age of the participants varied from 6.6 years-old in the STARS cohort to 20.0 years-old in RAINE. The genetic risk score and 4 SNPs – rs7744813 (KCNQ5), rs7837791 (*TOX*), rs8000973 (*ZIC2*) and rs17648524 (*A2BP1*) – were associated with refractive error (P < 0.05; Table 3). All 4 SNPs had the expected direction of effect and none exhibited evidence of between-cohort heterogeneity. Interestingly, 3 of the 4 SNPs had evidence of both early-onset and later progressive effects in the discovery cohort. Meta-analysis summary plots for the genetic risk score and the individual SNPs tested for replication are presented in Figure S1. There was suggestive evidence that SNPs had larger effect sizes in Asian than in European ancestry participants (Figure S2).

Tests in the Discovery Cohort for SNP x SNP interactions for all 741 possible pairs of the 39 SNPs revealed no evidence for interactions exceeding that expected by chance (not shown).

### Interactions with time engaged in nearwork

Two types of interactions between SNP genotype and nearwork exposure were evaluated in the ALSPAC discovery cohort: An interaction already present at the baseline age of 7.5 years-old (a 2-way SNP x nearwork interaction) and an interaction that developed progressively during later childhood (a 3-way, SNP x nearwork x age-from-baseline interaction). For a SNP with a risk allele frequency of 0.25, and ignoring the repeated measures nature of the data, the analysis of ALSPAC participants had >90% power to detect an interaction effect of 0.25 D at α = 0.05 (and >50% power at α = 1.28E–3, corresponding to a Bonferroni correction for testing all 39 SNPs).

**Figure 3:**
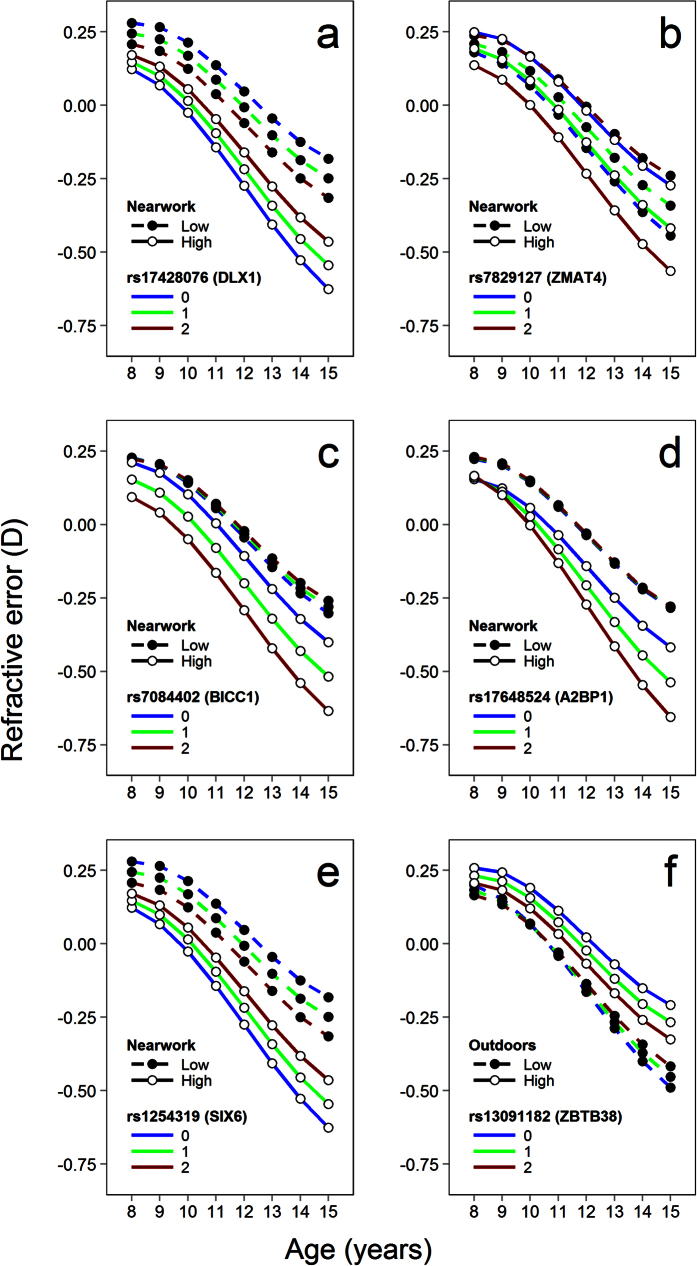
Refractive error trajectories in ALSPAC participants for SNPs showing evidence of an interaction with nearwork or time outdoors. Levels of nearwork activity and time spent outdoors were assessed at 8–9 years of age and classified as high or low (above or below the median level). Panels (**a–d**) show how refractive error trajectories varied depending on nearwork level and the number of risk alleles (0–2) carried for 4 different markers that showed SNP x nearwork x age-from-baseline (3-way) interactions. Panel (**e**) Refractive trajectories for the only marker to show a SNP x nearwork (2-way) interaction at baseline age. Panel (**f** ) Refractive trajectories for the only marker to show a SNP x time outdoors x age-from-baseline (3-way) interaction. The coloured lines show the trajectories predicted by the best-fit linear mixed model for children carrying the number of copies of the risk allele indicated in the legend.

Nominal support for 3-way SNP x nearwork x age-from-baseline interactions was observed for 4 markers (Fig. 3a–d): rs17428076 upstream of *DLX1* (P = 0.049), rs7829127 within *ZMAT4* (P = 6.3E–04), rs7084402 upstream of *BICC1* (P = 0.043) and rs17648524 within *A2BP1* (P = 2.3E–03). In models that considered just 2-way interactions at baseline, only rs1254319 upstream of *SIX6* showed nominal evidence of an interaction (P = 0.042; Fig. 3e). Of these 5 interactions, only that involving rs7829127 (*ZMAT4*) survived correction for multiple testing (corrected P = 0.025). Consistent with the limited evidence for individual SNP x nearwork interactions, no evidence of interaction between the genetic risk score and ALSPAC children’s level of nearwork was observed (2-way interaction, P = 0.20; 3-way interaction, P = 0.086).

Four of the cross-sectional study cohorts, 1 of European ancestry (TEDS) and 3 of Asian ancestry (GZT, SCORM and STARS), had information available regarding the time participants spent engaged in nearwork (Table S6), allowing tests for replication. In the meta-analysis of all 4 replication studies (Table S3) none of the SNPs that showed nominal evidence of an interaction with nearwork in the ALSPAC discovery cohort showed evidence of replication (all P > 0.16). Likewise, the genetic risk score did not show evidence of an interaction with nearwork in the cross-sectional cohorts (P = 0.49).

### Interactions with time spent outdoors

In the discovery cohort, only rs13091182 within *ZBTB38* showed nominal evidence of a 3-way interaction involving time outdoors (uncorrected P = 0.028; corrected P > 0.05; Fig. 3f). Surprisingly, the risk allele of rs13091182 was associated with *slower* progression towards myopia (or less hyperopia) in general and with faster progression towards myopia in children who spent *more* time outdoors, suggesting a potentially false-positive result. There was no evidence for 2-way SNP x time outdoors interactions (uncorrected P > 0.20 for all 39 SNPs). Similarly, for the genetic risk score, there was no indication of an interaction with time spent outdoors (2-way interaction, P = 0.16; 3-way interaction, P = 0.49).

Five of the cross-sectional samples had information available on the time participants spent outdoors (TEDS, RAINE, GZT, SCORM and STARS). The single SNP, rs13091182, showing evidence of an interaction with time outdoors in the discovery cohort showed no evidence of replication (indeed, none of the 31 SNPs with MAF > 0.05 in both ancestry groups showed evidence of an interaction with time outdoors; all P > 0.17; Table S4). Similarly, the genetic risk score did not show evidence of an interaction with time spent outdoors in the replication cohorts.

### Pathway analysis

Pathway analysis identified a single functional pathway for the set of 10 genes (Table 2) implicated in having early-onset effects, namely “*hedgehog signalling*” (Panther P = 0.043; key genes *ZIC2* and *BMP4*). The set of 11 genes implicated in having later-onset effects did not show enrichment for specific pathways.

## Discussion

### Early-onset and later-onset SNP effects

Sixteen SNPs showed evidence of effects in childhood in ALSPAC participants (Table 2); 10 SNPs had early-onset effects manifest by age 7.5 years, 11 SNPs had later-onset effects, and 5 SNP had early-onset effects that progressed further during later childhood. For the 12 of these 16 SNPs available in the cross-sectional cohorts, 4 showed evidence of replication (Table 3). There was suggestive evidence that SNP effect sizes were approximately 2 times larger in Asian as compared to European ancestry children/adolescents (Figure S2). A genetic risk score that captured the effects of all 39 GWAS-identified variants confirmed the involvement of genetic influences acting at an early age (7.5 years) and then increasing further in magnitude across later childhood.

We sought to discover whether the early-onset and later-onset variants clustered according to functional pathway (for example, if GWAS SNPs A and B are causal variants that affect the expression levels of genes X and Y, respectively, and X acts downstream of Y to regulate refractive development, then one might expect the onset age for SNPs A and B to coincide). However, as summarised in Table 4, SNPs associated with early-onset or later-onset effects did not clearly cluster according to the known function(s) of the genes implicated in mediating the SNPs’ effects. Pathway analysis confirmed this impression, with only a single functional pathway being identified. Potential reasons for this lack of functional clustering are, first, that many genes in the genome have diverse functions, which are sometimes poorly understood. For instance, during development of the human visual system, an ion channel may play a vital role during early embryonic development of the retina, be a necessary component of the visual cycle, and yet also contribute to neuronal plasticity. Second, precisely which gene or genes mediate the effect of a specific GWAS-identified SNP is not known with certainty for any of the refractive error GWAS SNPs identified to date: While the nearest gene to a GWAS SNP is usually considered the most likely to be involved, this does not always hold true^35^.

The 39 SNPs examined were identified in adult GWAS meta-analyses with sample sizes of approximately 45,000 individuals, and all had small effects (typically 0.1 D per copy of the risk allele). The ALSPAC longitudinal cohort (N = 5,200) had ~80% power to detect an association for a SNP with an effect size of 0.1 D and MAF of 0.25 (but note that the true power would likely have been lower because: refractive development would not be complete by 15 years of age, our models tested primarily for yearly effects rather than cumulative effects, and the “winner’s curse” phenomenon^36^, i.e. the over-estimation of effect sizes in the original GWAS investigations). Therefore, a likely reason why some of the 39 SNPs we studied failed to show childhood-onset associations in the longitudinal cohort is limited statistical power. Thus, we *cannot* conclude that the SNPs that did not show observable childhood-onset associations have an age-of-onset beyond 15 years-old even though they might well do: much larger studies will be required to definitively address this issue. Similarly, the limited concordance between the longitudinal and cross-sectional studies was also likely due to limited statistical power, although 8 of the 12 SNPs tested for replication showed the expected direction of effect (Table 3).

### Interactions with environmental exposures

In general there was scant evidence for GxE interactions, especially for SNP x time spent outdoors effects. Given the expected power of >90% to detect interaction effects with a magnitude 0.25 D or more, this argues against SNP x nearwork or SNP x time outdoors interactions of this size being present for the majority of variants studied, rather than lack of statistical power precluding their discovery.

In the ALSPAC longitudinal analysis the gene-environment interaction between *ZMAT4* SNP rs7829127 genotype and nearwork survived correction for multiple-testing (P_corr_ = 0.025). Although this interaction was not replicated in the cross-sectional meta-analyses, variants at this locus have previously been reported to show an interaction with the duration of education in a meta-analysis of 5 studies from Singapore (SNP x education interaction = −0.42 D, 95% C.I. −0.15 to −0.69, P = 0.002)^37^. We did not explore interactions between SNPs and years of education, since in several cohorts the participants were still students. The functional role of *ZMAT4* is not known.

Why might GxE interactions involving these 39 SNPs be so scarce? First, differences in environmental risk exposures were not considered in the original GWAS investigations carried out by CREAM^9^ and 23andMe^8^. Thus, SNPs with strong interaction effects but no main effects may not have been detected using those GWAS designs. Second, the age range and ethnic diversity of the original GWAS discovery samples were highly varied. Given the substantial increase in the prevalence of myopia in the past few decades, which strongly implicates a major role for environmental risk factors, it seems almost certain that the individuals studied in the CREAM and 23andMe GWAS meta-analyses would have grown up in environments with a wide range of risk exposure profiles depending on the participants’ years of birth: young (recently born) individuals would have been exposed to a much more myopiagenic environment than older (more distantly born) adults. Therefore, a variant that increases the risk of myopia only in children who perform excessive nearwork may have shown an (apparent) main effect association with refractive error in a GWAS carried out in a young adult cohort, in which participants were ubiquitously exposed to high nearwork during childhood. However, this same variant may not have shown an association with refractive error in a GWAS on an older cohort, due to the lower nearwork exposure during childhood of the older individuals. Thus, support for the association of such a variant in the CREAM and 23andMe GWAS samples may have been diluted rather than strengthened during the meta-analysis of younger and older cohorts.

Separate from tests for gene-environment interactions, time spent outdoors itself *was not* associated with myopia in 3 of the 5 cross-sectional studies (GZT, STARS, and TEDS) and the association was of borderline significance in another (TEDS). This lack of an association with time outdoors implies that detecting a SNP x time outdoors interaction would also have been challenging, even after meta-analysis of data from all 5 cohorts.

Interestingly, a large-effect GxE interaction predisoposing children to myopia was identified recently, involving a rare variant at the *APLP2* gene locus and time spent reading^38^. *APLP2* was implicated in myopia development through studies in an animal model^39^, which – given the statistical challenge of identifying GxE interaction effects in human populations – suggests that combining findings from animal models and human studies could be a fruitful future approach.

We reasoned that correction for multiple testing *was not* appropriate when examining the age-of-onset of the 39 SNPs investigated, because of compelling existing evidence that by adulthood these SNPs truly are associated with refractive error. That is, our analyses sought to discover whether or not each SNP had an effect during childhood, not whether a group of candidate SNPs were associated with refractive error *per se*. By contrast, in view of very limited evidence for interactions with environmental exposures for most of the SNPs examined, correction for multiple testing *was* considered appropriate when evaluating SNP x nearwork and SNP x time outdoors interactions: In these analyses, a large number of independent hypothesis tests were carried out, with little or no prior knowledge that an interaction must be present at some age.

### Limitations of the present work

The present work had a number of other limitations. The cross-sectional samples were not matched for age, which prevented us from testing for “early” and “later” onset effects in the replication stage. The level of exposure to nearwork and time outdoors also varied across samples, which meant that imprecisely-matched interaction effects were meta-analysed, potentially reducing statistical power. We chose to categorise time spent performing nearwork and time spent outdoors relative to the median activity level in each study sample because the measurement scales used in the various studies were not standardised (precluding the use of an absolute measure). If in reality these environmental risk factors exert their influence non-linearly – for instance if spending more than a certain threshold number of hours per day outdoors is needed to protect against myopia development – then our approach may have poorly captured the effects of the environmental exposures. For the combined meta-analysis of European and Asian cross-sectional studies, we assumed that each lead SNP tagged the underlying causal variant(s) equally well in European and Asian ancestry individuals, which is an oversimplification. Finally, we chose to examine only a simple, binary GxE model, whereas more complex scenarios may exist^40^,^41^,^42^.

## Conclusions

Specific myopia-predisposing SNPs were found to differ in the age at which they had their effects, and whether or not these effects got progressively stronger during later childhood. Thus, SNPs implicating the genes *CHRNG*, *CACNA1D*, *LAMA2*, *CYP26A1* and *BMP4* were associated with early onset changes in refractive error that did not progress further, while SNPs close to *PRSS56*, *KCNQ5*, *TOX*, *ZIC2 and SHISA6* showed early-onset effects that became greater still at older ages. Effects that only appeared in later childhood – after the age of 7.5 years – implicated the genes *CD55, CHD7*, *RORB*, *KCNMA1*, *A2BP1* and *GJD2*. Gene-environment interactions involving nearwork or time outdoors were rare or absent for the vast majority of the GWAS-identified SNPs, and indeed a genetic risk score that demonstrated very convincing association with early-onset (P = 2.2E–9) and later progressive (P = 5.8E–14) changes in refractive error appeared to act independently of the time children spent in these activities. However, one robust interaction between rs7829127 in *ZMAT4* and time spent performing nearwork (nominal P = 6.3E–04, corrected P = 0.025) was observed, replicating a previously-identified interaction involving rs7829127 and years of education^37^,^43^,^44^.

## Additional Information

**How to cite this article**: Fan, Q. *et al.* Childhood gene-environment interactions and age-dependent effects of genetic variants associated with refractive error and myopia: The CREAM Consortium. *Sci. Rep.* **6**, 25853; doi: 10.1038/srep25853 (2016).

## Supplementary Material

Supplementary Information

## Figures and Tables

**Table 1:** Demographics of study samples.

Longitudinal cohort (N = 5,200)					
Study	Ethnicity	N	Female (%)	Age-at-baseline	Years follow-up
ALSPAC*	European	5200	51.0	7.5 (0.3)	7.0 (1.5)
**Cross-sectional cohorts (N = 5,599)**					
**Study**	**Ethnicity**	**N**	**Female (%)**	**Age (years)**	**Refraction (D)**
TEDS	European	698	56.0	16.2 (1.8)	−0.38 (1.70)
WESDR	European	289	50.5	17.7 (4.6)	−1.09 (1.79)
TEST	European	410	57.2	11.8 (5.0)	0.36 (1.24)
RAINE	European	754	50.9	20.0 (0.4)	−0.06 (1.53)
BATS	European	992	53.6	19.1 (3.2)	−0.33 (1.42)
GZT	Asian	1055	51.8	15.6 (2.8)	−1.97 (2.49)
SCORM	Asian	994	48.4	7.5 (0.9)	−0.55 (1.73)
STARS	Asian	407	49.4	6.6 (3.9)	−2.00 (2.09)

Values in brackets are standard deviations.

^*^Refraction details at each age for the longitudinal cohort are provided in the supplementary material (Table S8).

**Table 2:** Age-of-onset of SNP associations with refractive error in the discovery cohort (ALSPAC).

					SNP main effect at baseline (D)			SNP x Age interaction (D/yr)		
Marker	Chr	Gene	RA	RAF	Beta	SE	P	Beta	SE	P
GR Score	–	–	–	–	**−0.018**	**0.003**	**2.2E**–**09**	**−0.003**	**0.000**	**5.8E**–**14**
rs1652333	1	CD55	G	0.32	−0.002	0.019	9.3E–01	**−0.005**	**0.003**	**4.0E**–**02**
rs1656404	2	PRSS56	A	0.21	**−0.066**	**0.024**	**5.7E**–**03**	**−0.008**	**0.003**	**1.3E**–**02**
rs1881492	2	CHRNG	T	0.23	**−0.058**	**0.024**	**1.7E**–**02**	−0.005	0.003	1.5E–01
rs14165	3	CACNA1D	G	0.70	**−0.040**	**0.020**	**4.2E**–**02**	−0.001	0.003	7.7E–01
rs7744813	6	KCNQ5	A	0.59	**−0.048**	**0.019**	**9.9E**–**03**	**−0.005**	**0.003**	**3.5E**–**02**
rs12205363	6	LAMA2	T	0.92	**−0.097**	**0.035**	**5.7E**–**03**	−0.008	0.005	1.2E–01
rs7837791	8	TOX	G	0.53	**−0.045**	**0.018**	**1.1E**–**02**	**−0.005**	**0.002**	**2.7E**–**02**
rs4237036	8	CHD7	T	0.66	0.020	0.019	2.9E–01	**−0.007**	**0.003**	**5.6E**–**03**
rs7042950	9	RORB	G	0.22	0.018	0.022	4.1E–01	**−0.009**	**0.003**	**2.5E**–**03**
rs6480859	10	KCNMA1	T	0.37	−0.029	0.018	1.1E–01	**−0.008**	**0.002**	**1.3E**–**03**
rs10882165	10	CYP26A1	T	0.40	**−0.035**	**0.018**	**4.8E**–**02**	0.001	0.003	7.6E–01
rs8000973	13	ZIC2	C	0.52	**−0.042**	**0.018**	**1.8E**–**02**	**−0.008**	**0.002**	**1.5E**–**03**
rs66913363	14	BMP4	G	0.51	**−0.051**	**0.018**	**5.3E**–**03**	0.001	0.003	7.2E–01
rs524952	15	GJD2	A	0.46	−0.018	0.018	3.3E–01	**−0.008**	**0.003**	**8.8E**–**04**
rs17648524	16	A2BP1	C	0.33	−0.001	0.019	9.4E–01	**−0.007**	**0.003**	**5.6E**–**03**
rs2969180	17	SHISA6	A	0.35	**−0.039**	**0.019**	**3.9E**–**02**	**−0.005**	**0.003**	**4.9E**–**02**

Abbreviations: Chr = Chromosome. GR = Genetic risk. RA = Risk allele. RAF = Risk allele frequency.

Associations were tested at baseline (age of 7.5 years-old) and over the next 7 years (SNP x Age interaction). Results for all 39 SNPs are shown in Table S2.

**Table 3:** Replication meta-analysis results for SNP main effects.

				Europeans (N = 3,143)				Asians (N = 2,456)				Europeans + Asians (N = 5,599)				
Marker	Chr	Gene	RA	*RAF*	Beta	SE	P	*RAF* >*	Beta	SE	P	I^2^	Het_P	Beta	SE	P
GR Score	–	–	–	–	**−0.026**	**0.007**	**3.8E**–**04**	–	**−0.048**	**0.011**	**1.4E**–**05**	0.57	**0.023**	−**0.034**	**0.006**	**1.4E**–**08**
rs1652333	1	*CD55*	G	0.32	0.042	0.042	0.315	0.52	–0.101	0.056	0.073	0.27	0.210	−0.004	0.034	0.899
rs1881492	2	*CHRNG*	T	0.23	−0.001	0.054	0.986	0.12	0.197	0.102	0.054	0.00	0.926	0.033	0.048	0.483
rs7744813	6	*KCNQ5*	A	0.59	−**0.110**	**0.042**	**0.008**	0.81	0.001	0.071	0.993	0.41	0.107	−**0.083**	**0.036**	**0.021**
rs7837791	8	*TOX*	G	0.53	0.011	0.040	0.772	0.53	−**0.185**	**0.055**	**0.001**	0.49	0.059	−**0.063**	**0.032**	**0.049**
rs4237036	8	*CHD7*	T	0.66	−0.077	0.041	0.062	0.74	0.102	0.069	0.140	0.40	0.112	−0.033	0.035	0.358
rs7042950	9	*RORB*	G	0.22	0.041	0.047	0.391	0.74	−0.004	0.070	0.956	0.00	0.903	0.020	0.039	0.618
rs6480859	10	*KCNMA1*	T	0.37	−0.022	0.041	0.579	0.16	−**0.229**	**0.074**	**0.002**	0.52	0.042	−0.063	0.036	0.075
rs8000973	13	*ZIC2*	C	0.52	−0.067	0.040	0.093	0.21	−0.092	0.070	0.190	0.00	0.470	−**0.081**	**0.035**	**0.019**
rs66913363	14	*BMP4*	G	0.51	−0.021	0.044	0.628	0.73	0.061	0.066	0.354	0.00	0.790	0.002	0.037	0.953
rs524952	15	*GJD2*	A	0.46	−0.008	0.041	0.839	0.48	−**0.171**	**0.057**	**0.003**	0.53	**0.036**	−0.064	0.033	0.058
rs17648524	16	*A2BP1*	C	0.33	−**0.143**	**0.042**	**7.2E**–**04**	0.06	−0.140	0.106	0.186	0.49	0.057	−**0.146**	**0.039**	**2.0E**–**04**
rs2969180	17	*SHISA6*	A	0.35	0.028	0.042	0.499	0.51	−0.036	0.056	0.521	0.00	0.553	0.003	0.033	0.926

SNPs associated with refractive error in the ALSPAC age-of-onset analyses were tested for association with refractive error in 8 independent cohorts of children (5 European ancestry, 3 Asian ancestry).

Abbreviations: Chr = Chromosome. GR Score = Genetic risk score. RA = Risk allele. RAF = Risk allele frequency. ^*^SNPs with minor allele frequencies <0.05 were not examined due to low statistical power.

**Table 4:** Summary of findings.

SNP	Gene	Role	Longitudinal Early-onset	Longitudinal Later-onset	Cross-sectional	Interaction
GR score	–	–	Y	Y	Y	
rs7837791	TOX	ED	Y	Y	Y	
rs4237036	CHD7	ED		Y		
rs7084402	BICC1	ED				NW
rs8000973	ZIC2	ED	Y	Y	Y	
rs66913363	BMP4	ED	Y			
rs1254319	SIX6	ED				NW
rs1656404	PRSS56	ED, EM	Y	Y		
rs17428076	DLX1	ED,NP				NW
rs12205363	LAMA2	EM	Y			
rs1652333	CD55	IT		Y		
rs1881492	CHRNG	IT	Y			
rs14165	CACNA1D	IT	Y			
rs6480859	KCNMA1	IT		Y		
rs7744813	KCNQ5	IT, VC	Y	Y	Y	
rs17648524	A2BP1	NP		Y	Y	NW
rs13091182	ZBTB38	U				TO
rs9307551	LOC100506035	U				NW
rs7829127	ZMAT4	U				NW
rs2969180	SHISA6	U	Y	Y		
rs7042950	RORB	VC		Y		
rs10882165	CYP26A1	VC	Y			
rs524952	GJD2	VC		Y		

SNPs with evidence (P < 0.05) of early-onset, later onset, or GxE interaction effects on refractive error in one or more analysis are highlighted.

Abbreviations: Y = Yes, NW = Nearwork, TO = Time outdoors, VC = Visual cycle, NP = Neuronal plasticity, IT = Ion transport, EM = Extracellular matrix, ED = Eye development, U = Unknown.
