# Supplementary material for: Childhood gene-environment interactions and age-dependent effects of genetic variants associated with refractive error and myopia: The CREAM Consortium
Source: Sci Rep. 2016 May 13;6:25853. doi: 10.1038/srep25853 (PMC4865831; doi:10.1038/srep25853)
Supplement: Supplementary Information [file srep25853-s1.pdf]

Supplementary Information

Childhood gene-environment interactions and age-dependent effects of genetic variants associated with refractive error and myopia: The CREAM Consortium

Authors

Qiao Fan[1*], Xiaobo Guo[2,3*], J. Willem L. Tideman[4,5*], Katie M Williams[6,7], Seyhan Yazar[8], Mohsen Hosseini[9], Laura D. Howe[10,11], Beaté St Pourcain[10,12], David M. Evans[10,13], Nicholas J. Timpson[10], George McMahon[10], Pirro G. Hysi[7], Eva Krapohl[14], Ya Xing Wang[15,16], Jost B. Jonas[15,16,17], Paul N. Baird[18], Jie Jin Wang[18,19], Ching-Yu Cheng[1,20,21], Yik-Ying Teo[21,22], Tien-Yin Wong[1,20], Xiaohu Ding[3], Robert Wojciechowski[23,24], Terri L. Young[25,26], Olavi Pärssinen[27,28], Konrad Oexle[29], Norbert Pfeiffer[30], Joan E. Bailey-Wilson[23], Andrew D. Paterson[9], Caroline C. W. Klaver[4,5], Robert Plomin[14], Christopher J. Hammond[6,7†], David A. Mackey[8,18†], Mingguang He[3,18†], Seang-Mei Saw[1,21†], Cathy Williams[11†], Jeremy A. Guggenheim[31†], The CREAM Consortium.

Affiliations

[1]Singapore Eye Research Institute, Singapore National Eye Centre, Singapore, Singapore. [2]Department of Statistical Science, School of Mathematics & Computational Science, Sun Yat-Sen University, Guangzhou. [3]State Key Laboratory of Ophthalmology, Zhongshan Ophthalmic Center, Sun Yat-sen University, Guangzhou, China. [4]Department of Epidemiology, Erasmus Medical Center, Rotterdam, The Netherlands. [5]Department of Ophthalmology, Erasmus Medical Center, Rotterdam, The Netherlands. [6]Department of Ophthalmology, King's College London, St Thomas' Hospital campus, London, UK. [7]Department of Twin Research and Genetic Epidemiology, King's College London School of Medicine, London, UK. [8]Centre for Ophthalmology and Visual Science, Lions Eye Institute, University of Western Australia, Perth, Australia. [9]Genetics and Genome Biology Program, Hospital for Sick Children and University of Toronto, Toronto, Ontario, Canada. [10]MRC Integrative Epidemiology Unit (IEU) at the University of Bristol, Bristol, UK. . [11]School of Social and Community Medicine, University of Bristol, Bristol, UK. [12]Max Planck Institute for Psycholinguistics, Wundtlaan 1, 6525 XD Nijmegen, The Netherlands. [13]University of Queensland Diamantina Institute, Translational Research Institute, Brisbane, Queensland, Australia. [14]MRC Social, Genetic and Developmental Psychiatry Centre, Institute of Psychiatry, Psychology & Neuroscience, King's College London, London, UK. [15]Beijing Institute of Ophthalmology, Beijing Tongren Hospital, Capital Medical University, Beijing, China. [16]Beijing Ophthalmology and Visual Science Key Lab, Beijing, China. [17]Department of Ophthalmology, Medical Faculty Mannheim, Ruprecht-Karls-University Heidelberg, Mannheim, Germany. [18]Centre for Eye Research Australia (CERA), University of Melbourne, Royal Victorian Eye and Ear Hospital, Melbourne, Victoria, Australia. [19]Centre for Vision Research, Department of Ophthalmology and Westmead Millennium Institute of Medical Research, University of Sydney, Sydney, Australia. [20]Department of Ophthalmology, National University Health Systems, National University of Singapore, Singapore. [21]Saw Swee Hock School of Public Health, National University Health Systems, National University of Singapore, Singapore, Singapore. [22]Department of Statistics and Applied Probability, National University of Singapore, Singapore, Singapore. [23]Computational and Statistical Genomics Branch, National Human Genome Research Institute, National Institutes of Health, Baltimore, MD, USA. [24]Department of Epidemiology, Johns Hopkins Bloomberg School of Public Health, Baltimore, Maryland, USA. [25]Ophthalmology and Visual Sciences, University of Wisconsin-Madison, Madison, WI, USA. [26]Duke-National University of Singapore Graduate Medical School, Singapore, Singapore. [27]Department of Health Sciences and Gerontology Research Center, University of Jyväskylä, Jyväskylä, Finland. [28]Department of Ophthalmology, Central Hospital of Central Finland, Jyväskylä, Finland. [29]Institute of Human Genetics, Technical University Munich, Munich, Germany. [30]Department of Ophthalmology, University Medical Center Mainz, Mainz,

Germany. [31]School of Optometry & Vision Sciences, Cardiff University, Cardiff, UK. [*]These authors contributed equally to this work. [†]These authors jointly led this work.

Index

Table S1. SNPs examined.	3
Table S2. Age-of-onset of SNP associations in Discovery cohort (ALSPAC).	4
Table S3. Meta-analysis of SNP x near work interaction effects in cross-sectional cohorts.	5
Table S4. Meta-analysis of SNP x time outdoors interaction effects in cross-sectional cohorts.	6
Table S5. Genotyping and imputation details.	7
Table S6. Near work exposure in each cohort.	8
Table S7. Time outdoors exposure in each cohort.	9
Table S8. Refraction details for the ALSPAC discovery cohort.	10
Figure S1. Meta-analysis summary plots for cross-sectional cohorts.	11
Figure S2. SNP effects in European and Asian meta-analysis samples.	15
Figure S3. SNP x nearwork interactions at ages 7 and 12 in the ALSPAC discovery cohort.	16
Recruitment of participants and phenotypic assessment.	17
Supplementary references	22

Table S1. SNPs examined.

SNP	Chr	Pos	Gene	Citation
rs1652333	1	207470460	*CD55*	Verhoeven et al. 2013
rs4373767	1	219759682	*ZC3H11B*	Cheng et al. 2013
rs17412774	2	146773948	*PABPCP2*	Kiefer et al. 2013
rs17428076	2	172851936	*DLX1*	Kiefer et al. 2013
rs1898585	2	178660450	*PDE11A*	Kiefer et al. 2013
rs1656404	2	233379941	*PRSS56*	Verhoeven et al. 2013
rs1881492	2	233406998	*CHRNG*	Verhoeven et al. 2013
rs14165	3	53847408	*CACNA1D*	Verhoeven et al. 2013
rs13091182	3	141133960	*ZBTB38*	Kiefer et al. 2013
rs9307551	4	80530671	*LOC100506035*	Verhoeven et al. 2013
rs5022942	4	81959966	*BMP3*	Kiefer et al. 2013
rs7744813	6	73643289	*KCNQ5*	Verhoeven et al. 2013
rs12205363	6	129834628	*LAMA2*	Verhoeven et al. 2013
rs7829127	8	40726394	*ZMAT4*	Verhoeven et al. 2013
rs7837791	8	60179086	*TOX*	Verhoeven et al. 2013
rs4237036	8	61701057	*CHD7*	Verhoeven et al. 2013
rs11145465	9	70989531	*TJP2*	Verhoeven et al. 2013
rs7042950	9	77149837	*RORB*	Verhoeven et al. 2013
rs7084402	10	60265404	*BICC1*	Verhoeven et al. 2013
rs6480859	10	79081948	*KCNMA1*	Kiefer et al. 2013
rs745480	10	85986554	*RGR*	Kiefer et al. 2013
rs10882165	10	94924324	*CYP26A1*	Verhoeven et al. 2013
rs1381566	11	40149607	*LRRC4C*	Kiefer et al. 2013
rs2155413	11	84634790	*DLG2*	Kiefer et al. 2013
rs11601239	11	105556598	*GRIA4*	Verhoeven et al. 2013
rs3138144	12	56114768	*RDH5*	Verhoeven et al. 2013
rs12229663	12	71249996	*PTPRR*	Verhoeven et al. 2013
rs8000973	13	100691367	*ZIC2*	Verhoeven et al. 2013
rs2184971	13	100818092	*PCCA*	Verhoeven et al. 2013
rs66913363	14	54413001	*BMP4*	Kiefer et al. 2013
rs1254319	14	60903757	*SIX6*	Verhoeven et al. 2013
rs524952	15	35005885	*GJD2*	Verhoeven et al. 2013
rs4778879	15	79372875	*RASGRF1*	Verhoeven et al. 2013
rs17648524	16	7459683	*A2BP1*	Verhoeven et al. 2013
rs2969180	17	11407901	*SHISA6*	Verhoeven et al. 2013
rs17183295	17	31078272	*MYO1D*	Verhoeven et al. 2013
rs4793501	17	68718734	*KCNJ2*	Verhoeven et al. 2013
rs12971120	18	72174023	*CNDP2*	Verhoeven et al. 2013
rs235770	20	6761765	*BMP2*	Verhoeven et al. 2013

Table S2. Age-of-onset of SNP associations in discovery cohort (ALSPAC).

					SNP main effect at baseline (D)			SNP x Age interaction (D/yr)		
**Marker**	**Chr**	**Gene**	**RA**	**RAF**	**Beta**	**SE**	**P**	**Beta**	**SE**	**P**
GR Score	-	-	-	-	**-0.018**	**0.003**	**2.2E-09**	**-0.003**	**0.000**	**5.8E-14**
rs1652333	1	*CD55*	G	0.32	-0.002	0.019	9.3E-01	**-0.005**	**0.003**	**4.0E-02**
rs4373767	1	*ZC3H11B*	T	0.38	-0.005	0.018	8.0E-01	-0.001	0.003	7.9E-01
rs17412774	2	*PABPCP2*	A	0.57	-0.026	0.018	1.5E-01	-0.004	0.003	1.7E-01
rs17428076	2	*DLX1*	C	0.74	-0.026	0.021	2.1E-01	0.000	0.003	8.7E-01
rs1898585	2	*PDE11A*	T	0.17	0.005	0.025	8.3E-01	-0.006	0.003	1.1E-01
rs1656404	2	*PRSS56*	A	0.21	**-0.066**	**0.024**	**5.7E-03**	**-0.008**	**0.003**	**1.3E-02**
rs1881492	2	*CHRNG*	T	0.23	**-0.058**	**0.024**	**1.7E-02**	-0.005	0.003	1.5E-01
rs14165	3	*CACNA1D*	G	0.70	**-0.040**	**0.020**	**4.2E-02**	-0.001	0.003	7.7E-01
rs13091182	3	*ZBTB38*	G	0.67	-0.032	0.019	8.4E-02	0.001	0.003	6.4E-01
rs9307551	4	*LOC100506035*	A	0.20	-0.026	0.022	2.4E-01	-0.005	0.003	1.3E-01
rs5022942	4	*BMP3*	A	0.22	-0.003	0.021	8.7E-01	-0.004	0.003	1.8E-01
rs7744813	6	*KCNQ5*	A	0.59	**-0.048**	**0.019**	**9.9E-03**	**-0.005**	**0.003**	**3.5E-02**
rs12205363	6	*LAMA2*	T	0.92	**-0.097**	**0.035**	**5.7E-03**	-0.008	0.005	1.2E-01
rs7829127	8	*ZMAT4*	A	0.75	-0.006	0.022	7.7E-01	0.002	0.003	4.2E-01
rs7837791	8	*TOX*	G	0.53	**-0.045**	**0.018**	**1.1E-02**	**-0.005**	**0.002**	**2.7E-02**
rs4237036	8	*CHD7*	T	0.66	0.020	0.019	2.9E-01	**-0.007**	**0.003**	**5.6E-03**
rs11145465	9	*TJP2*	A	0.21	-0.036	0.021	9.6E-02	-0.004	0.003	2.4E-01
rs7042950	9	*RORB*	G	0.22	0.018	0.022	4.1E-01	**-0.009**	**0.003**	**2.5E-03**
rs7084402	10	*BICC1*	G	0.49	-0.019	0.018	3.0E-01	-0.001	0.003	7.7E-01
rs6480859	10	*KCNMA1*	T	0.37	-0.029	0.018	1.1E-01	**-0.008**	**0.002**	**1.3E-03**
rs745480	10	*RGR*	G	0.48	-0.021	0.018	2.3E-01	-0.003	0.002	2.6E-01
rs10882165	10	*CYP26A1*	T	0.40	**-0.035**	**0.018**	**4.8E-02**	0.001	0.003	7.6E-01
rs1381566	11	*LRRC4C*	G	0.18	-0.023	0.026	3.8E-01	-0.002	0.004	5.6E-01
rs2155413	11	*DLG2*	A	0.45	0.001	0.018	9.6E-01	0.000	0.002	9.8E-01
rs11601239	11	*GRIA4*	C	0.49	0.004	0.018	8.0E-01	-0.001	0.002	6.9E-01
rs3138144	12	*RDH5*	G	0.54	-0.027	0.021	1.9E-01	-0.002	0.003	5.2E-01
rs12229663	12	*PTPRR*	A	0.76	-0.033	0.022	1.3E-01	0.000	0.003	8.8E-01
rs8000973	13	*ZIC2*	C	0.52	**-0.042**	**0.018**	**1.8E-02**	**-0.008**	**0.002**	**1.5E-03**
rs2184971	13	*PCCA*	A	0.60	0.002	0.018	8.9E-01	0.000	0.002	9.1E-01
rs66913363	14	*BMP4*	G	0.51	**-0.051**	**0.018**	**5.3E-03**	0.001	0.003	7.2E-01
rs1254319	14	*SIX6*	A	0.29	-0.011	0.020	5.8E-01	-0.002	0.003	3.8E-01
rs524952	15	*GJD2*	A	0.46	-0.018	0.018	3.3E-01	**-0.008**	**0.003**	**8.8E-04**
rs4778879	15	*RASGRF1*	G	0.42	-0.017	0.018	3.7E-01	-0.004	0.003	9.4E-02
rs17648524	16	*A2BP1*	C	0.33	-0.001	0.019	9.4E-01	**-0.007**	**0.003**	**5.6E-03**
rs2969180	17	*SHISA6*	A	0.35	**-0.039**	**0.019**	**3.9E-02**	**-0.005**	**0.003**	**4.9E-02**
rs17183295	17	*MYO1D*	T	0.19	0.006	0.023	7.8E-01	-0.004	0.003	1.5E-01
rs4793501	17	*KCNJ2*	T	0.53	0.000	0.018	9.8E-01	-0.002	0.003	4.2E-01
rs12971120	18	*CNDP2*	A	0.82	0.017	0.021	4.1E-01	-0.003	0.003	3.2E-01
rs235770	20	*BMP2*	T	0.37	-0.010	0.019	5.8E-01	-0.005	0.003	5.3E-02

Abbreviations: Chr=Chromosome. RA=Risk allele. RAF=Risk allele frequency.

Table S3. Meta-analysis of SNP x near work interaction effects in cross-sectional cohorts. Beta shows the difference in refractive error (D) associated with each copy of the risk allele in individuals exposed to high versus low levels of nearwork. Meta-analysis was conducted for 4 cohorts (TEDS, GZT, SCORM and STARS) combined N=3,154.

SNP	Chr	Gene	RA	Beta	SE	P		I^2	$P_{Q\text{-test}}$
Allele score	-	-	A	-0.014	0.021	0.489		0	0.584
rs1652333	1	*CD55*	G	-0.049	0.108	0.649		0	0.460
rs4373767	1	*ZC3H11B*	T	-0.217	0.116	0.061		0	0.979
rs17412774	2	*PABPCP2*	A	0.157	0.114	0.169		0	0.877
rs1898585	2	*PDE11A*	T	-0.189	0.117	0.108		0	0.769
rs1881492	2	*CHRNG*	T	0.253	0.185	0.170		0	0.609
rs9307551	4	*LOC100506035*	A	**-0.237**	**0.113**	**0.035**		9	0.348
rs5022942	4	*BMP3*	A	-0.088	0.117	0.450		0	0.621
rs7744813	6	*KCNQ5*	A	0.251	0.134	0.061		0	0.856
rs7829127	8	*ZMAT4*	A	-0.104	0.166	0.529		55	0.084
rs7837791	8	*TOX*	G	-0.031	0.106	0.771		9	0.351
rs4237036	8	*CHD7*	T	-0.133	0.129	0.304		43	0.152
rs7042950	9	*RORB*	G	0.009	0.133	0.946		0	0.927
rs7084402	10	*BICC1*	G	-0.002	0.108	0.985		0	0.915
rs6480859	10	*KCNMA1*	T	-0.242	0.135	0.073		0	0.832
rs745480	10	*RGR*	G	0.020	0.109	0.854		0	0.712
rs1381566	11	*LRRC4C*	G	-0.060	0.129	0.644		0	0.502
rs2155413	11	*DLG2*	A	0.215	0.138	0.120		28	0.379
rs11601239	11	*GRIA4*	C	-0.008	0.111	0.943		0	0.765
rs3138144	12	*RDH5*	G	-0.083	0.170	0.625		0	0.409
rs12229663	12	*PTPRR*	A	0.042	0.111	0.704		0	0.832
rs8000973	13	*ZIC2*	C	-0.039	0.128	0.759		0	0.581
rs2184971	13	*PCCA*	A	0.091	0.127	0.473		0	0.896
rs66913363	14	*BMP4*	G	0.205	0.125	0.099		0	0.403
rs1254319	14	*SIX6*	A	-0.078	0.120	0.513		0	0.698
rs524952	15	*GJD2*	A	-0.033	0.110	0.761		15	0.317
rs4778879	15	*RASGRF1*	G	0.033	0.110	0.766		0	0.631
rs17648524	16	*A2BP1*	C	0.178	0.176	0.312		22	0.279
rs2969180	17	*SHISA6*	A	0.010	0.108	0.927		0	0.435
rs4793501	17	*KCNJ2*	T	0.047	0.110	0.671		56	0.078
rs12971120	18	*CNDP2*	A	-0.049	0.120	0.682		0	0.581
rs235770	20	*BMP2*	T	-0.031	0.131	0.814		0	0.847

Abbreviations: Chr=Chromosome. RA=Risk allele. I^2=Heterogeneity statistic. $P_{Q\text{-test}}$=P-value for Cochran's Q-test.

Table S4. Meta-analysis of SNP x time outdoors interaction effects in cross-sectional cohorts. Beta shows the difference in refractive error (D) associated with each copy of the risk allele in individuals exposed to high versus low levels of time outdoors. Meta-analysis was conducted for 5 cohorts (TEDS, RAINE, GZT, SCORM and STARS) combined N=3,908.

SNP	Chr	Gene	RA	Beta	SE	P		I^2	$P_{Q\text{-test}}$
Allele score	-	-	A	-0.003	0.019	0.892		29	0.231
rs1652333	1	*CD55*	G	0.108	0.104	0.301		2	0.394
rs4373767	1	*ZC3H11B*	T	0.132	0.104	0.202		0	0.974
rs17412774	2	*PABPCP2*	A	0.064	0.107	0.549		0	0.841
rs1898585	2	*PDE11A*	C	-0.038	0.120	0.754		0	0.706
rs1881492	2	*CHRNG*	G	0.011	0.156	0.946		48	0.101
rs9307551	4	*LOC100506035*	C	0.088	0.110	0.421		0	0.675
rs5022942	4	*BMP3*	G	0.028	0.114	0.804		0	0.550
rs7744813	6	*KCNQ5*	A	-0.097	0.116	0.404		8	0.361
rs7829127	8	*ZMAT4*	A	0.015	0.137	0.915		0	0.951
rs7837791	8	*TOX*	T	-0.032	0.099	0.746		0	0.528
rs4237036	8	*CHD7*	T	-0.081	0.114	0.477		0	0.927
rs7042950	9	*RORB*	A	0.101	0.122	0.411		0	0.708
rs7084402	10	*BICC1*	G	0.009	0.103	0.928		0	0.864
rs6480859	10	*KCNMA1*	C	-0.157	0.113	0.165		0	0.663
rs745480	10	*RGR*	C	-0.070	0.100	0.486		0	0.492
rs1381566	11	*LRRC4C*	T	-0.121	0.141	0.388		23	0.269
rs2155413	11	*DLG2*	A	-0.006	0.113	0.961		33	0.198
rs11601239	11	*GRIA4*	C	0.028	0.102	0.782		0	0.674
rs3138144	12	*RDH5*	G	-0.137	0.149	0.358		14	0.326
rs12229663	12	*PTPRR*	G	-0.045	0.109	0.681		0	0.587
rs8000973	13	*ZIC2*	T	-0.140	0.111	0.205		0	0.698
rs2184971	13	*PCCA*	G	-0.054	0.109	0.623		7	0.366
rs66913363	14	*BMP4*	G	0.016	0.122	0.896		0	0.703
rs1254319	14	*SIX6*	A	0.023	0.110	0.834		23	0.269
rs524952	15	*GJD2*	T	-0.055	0.106	0.606		0	0.829
rs4778879	15	*RASGRF1*	A	0.068	0.104	0.513		52	0.082
rs17648524	16	*A2BP1*	G	0.044	0.129	0.733		0	0.816
rs2969180	17	*SHISA6*	A	0.037	0.103	0.720		0	0.910
rs4793501	17	*KCNJ2*	C	-0.139	0.102	0.174		0	0.672
rs12971120	18	*CNDP2*	A	-0.027	0.116	0.813		6	0.372
rs235770	20	*BMP2*	C	-0.062	0.134	0.642		0	0.648

Abbreviations: Chr=Chromosome. RA=Risk allele. I^2=Heterogeneity statistic. $P_{Q\text{-test}}$=P-value for Cochran's Q-test.

Table S5. Genotyping and imputation details

Study	Genotyping platform	Imputation	Reference population (1000G)	QC
ALSPAC	Illumina HumanHap550	MACH/minimac	GIANT phase1 release v3	Cheng et al. 2013 [1]
BATS/TEST	Illumina HumanHap610/660-Quad	MACH	1000G Phase 1 release on Aug 4, 2010	Yazar et al. 2015 [2]
RAINE	Illumina 660W-Quad	MACH/minimac	1000G Phase 1 release on Nov 23, 2010	Yazar et al. 2015 [2]
TEDS	Affymetrix GeneChip 6.0	IMPUTE2 v2.3.0	1000G Phase 1 release v3	Davis et al. 2014 [3]
TEST	Illumina HumanHap610/660-Quad	MACH	1000G Phase 1 release on Aug 4, 2010	Yazar et al. 2015 [2]
WESDR	Illumina Human Omni1Quad	IMPUTE2 v2.3.0	1000G phase 1 integrated variant set release v3	Hosseini et al. 2015 [4]
Guangzhou Twins	Affymetrix Gene Titan	IMPUTE2 v2.3.0	1000 genomes phase 1 (Nov 2010 release)	-
SCORM	Illumina HumanHap550/550-Duo	MACH/minimac	1000 genomes phase 1 cosmopolitan panel haplotypes (March 2012 release)	Verhoeven et al. 2013
STARS Parents	Illumina HumanHap610-Quad	MACH/minimac	1000 genomes phase 1 cosmopolitan panel haplotypes (March 2012 release)	Verhoeven et al. 2013

Abbreviations: 1000G, One thousand genomes project. QC, Quality control.

Table S6. Time spent performing near work. Abbreviations: NA, Not available for analysis.

Cohort	Instrument	Low	High
ALSPAC	Maternal questionnaire: On normal days in school holidays, how much time on average does your child spend each day reading books for pleasure?<br>(a) None at all, (b) 1 hour, (c) 1–2 hours, (d) 3 or more hours.	<1.0 hrs/dy	≥1.0 hrs/dy
BATS	NA	NA	NA
GZT	Child questionnaire: How many hours per day do you spend doing near work in weekday?<br>How many hours per day do you spend doing near work in weekend?<br>During school terms (February to July, September to December), the average time of each type activity was calculated as (5×weekday + 2×weekend)/7. During holidays, the daily visual activity refers to weekend information. In China, every year has 9 months semester days and 3 months summer/winter holidays. The average nearwork per day in the past year was calculated as (9×semester day time + 3×holiday time)/12.	<4.2 hrs/dy	≥4.2 hrs/dy
RAINE	NA	NA	NA
SCORM	Maternal questionnaire:<br>Q1. In the past year, how many hours per day (outside school hours) did your child spend reading and writing?<br>(a) Weekdays: __ hours/day; (b) At the weekend: __hours/day.<br>Q2. In the past year, how many hours per day (outside of regular school hours) did your child spend watching TV, playing video games, and using a computer?<br>(a) Weekdays: __ hours/day; (b) At the weekend: __hours/day.<br><br>Total = (1a x 5/7)+(1b x 2/7)+(2a x 5/7)+(2b x 2/7)	<2.7 hrs/dy	≥2.7 hrs/dy
STARS	Maternal questionnaire:<br>Q1. During the school years, how many hours per day (outside of regular school hours) would you estimate your child spends reading and writing (school work & reading for pleasure?<br>(a) Weekdays: __ hours/day; (b) At the weekend: __hours/day.<br>Q2. During the school years, how many hours per day (outside of regular school hours) would you estimate your child spends drawing, watching TV, playing video games, computers, and other near work activity (cutting paper and playing toys etc)?<br>(a) Weekdays: __ hours/day; (b) At the weekend: __hours/day.<br>Total = (1a x 5/7)+(1b x 2/7)+(2a x 5/7)+(2b x 2/7)	<1.2 hrs/dy	≥1.2 hrs/dy
TEDS	Child questionnaire: Which of the following activities do you do, and how much do you enjoy them? If you have never had a go at these activities, please cross Never done.<br>(a) Reading for fun: _ hours per week<br>(b) Computer games: _hours per week<br>Total hours per day = (hours per week (a) + hours per week (b)) / 7	≤ 1.0 hrs/day	> 1.0 hrs/day
TEST	NA	NA	NA
WESDR	NA	NA	NA

Table S7. Time spent outdoors. Abbreviations: NA, Not available for analysis.

Cohort	Instrument	Low	High
ALSPAC	Maternal questionnaire: On a school weekday, how much time on average does your child spend each day out of doors in summer?<br>(a) None at all, (b) 1 hour, (c) 1–2 hours, (d) 3 or more hours.	<3.0 hrs/dy	≥3.0 hrs/dy
BATS	NA	NA	NA
GZT	Child questionnaire: How many hours per day do you spend outdoors in weekday?<br>How many hours per day do you spend outdoors in weekend?<br>During school terms (February to July, September to December), the average time of each type activity was calculated as (5×weekday + 2×weekend)/7. During holidays, the daily visual activity refers to weekend information. In China, every year has 9 months semester days and 3 months summer/winter holidays. The average nearwork per day in the past year was calculated as (9×semester day time + 3×holiday time)/12.	<1.4 hrs/dy	≥1.4 hrs/dy
RAINE	Young adult questionnaire: In the summer, when not working at your job or at school, what part of the day do you spend outside	≤1/4 of the day	>1/4 of the day
SCORM	Maternal questionnaire: How much time does your child spend outside:<br>(a) Plays outdoors (in the backyard, walks, bike riding): __ hours/day<br>(b) Participates in outdoor leisure activities (Family BBQs, park, Picnic, Beach): __ hours/day<br>(c) Outdoor sports: __ hours/day;<br>Total = (a) + (b) + (c)	<3.1 hrs/dy	≥3.1 hrs/dy
STARS	Maternal questionnaire: How much time does your child spend outside:<br>(a) Plays outdoors (in the backyard, walks, bike riding): __ hours/day<br>(b) Participates in outdoor leisure activities (Family BBQs, park, Picnic, Beach): __ hours/day<br>(c) Outdoor sports: __ hours/day;<br>Total = (a) + (b) + (c)	<0.5 hrs/dy	≥0.5 hrs/dy
TEDS	Child questionnaire: Which of the following activities do you do, and how much do you enjoy them? If you have never had a go at these activities, please cross Never done.<br>(a) Hang out with friends outside (eg, in park): _ hours per week<br><br>Total hours per day = total hours per week (a) / 7	≤0.6 hrs/day	> 0.6 hrs/day
TEST	NA	NA	NA
WESDR	NA	NA	NA

Table S8. Refraction details for the ALSPAC discovery cohort.

Clinic visit	N	Age (95% C.I.) in years	Refraction (95% C.I.) in D
7	4680	7.51 (7.50 to 7.52)	+0.18 (+0.16 to +0.21)
10	4955	10.63 (10.62 to 10.64)	+0.05 (+0.02 to +0.08)
11	4711	11.73 (11.72 to 11.74)	-0.04 (-0.07 to +0.00)
12	4740	12.80 (12.79 to 12.80)	-0.18 (-0.22 to -0.15)
15	3666	15.43 (15.42 to 15.44)	-0.39 (-0.43 to -0.35)

[Next page] **Figure S1. Meta-analysis summary plots for cross-sectional cohorts.** For each cohort, the change in refractive error per copy of the risk allele is shown by a black diamond (black horizontal line shows 95% confidence interval). The meta-analysis result is shown as a large diamond, with blue and red indicating meta-analysis $P \geq 0.05$ and $P < 0.05$, respectively. Note that SNPs with $MAF < 0.05$ in Asians were not analysed.

Figure S1 continued:

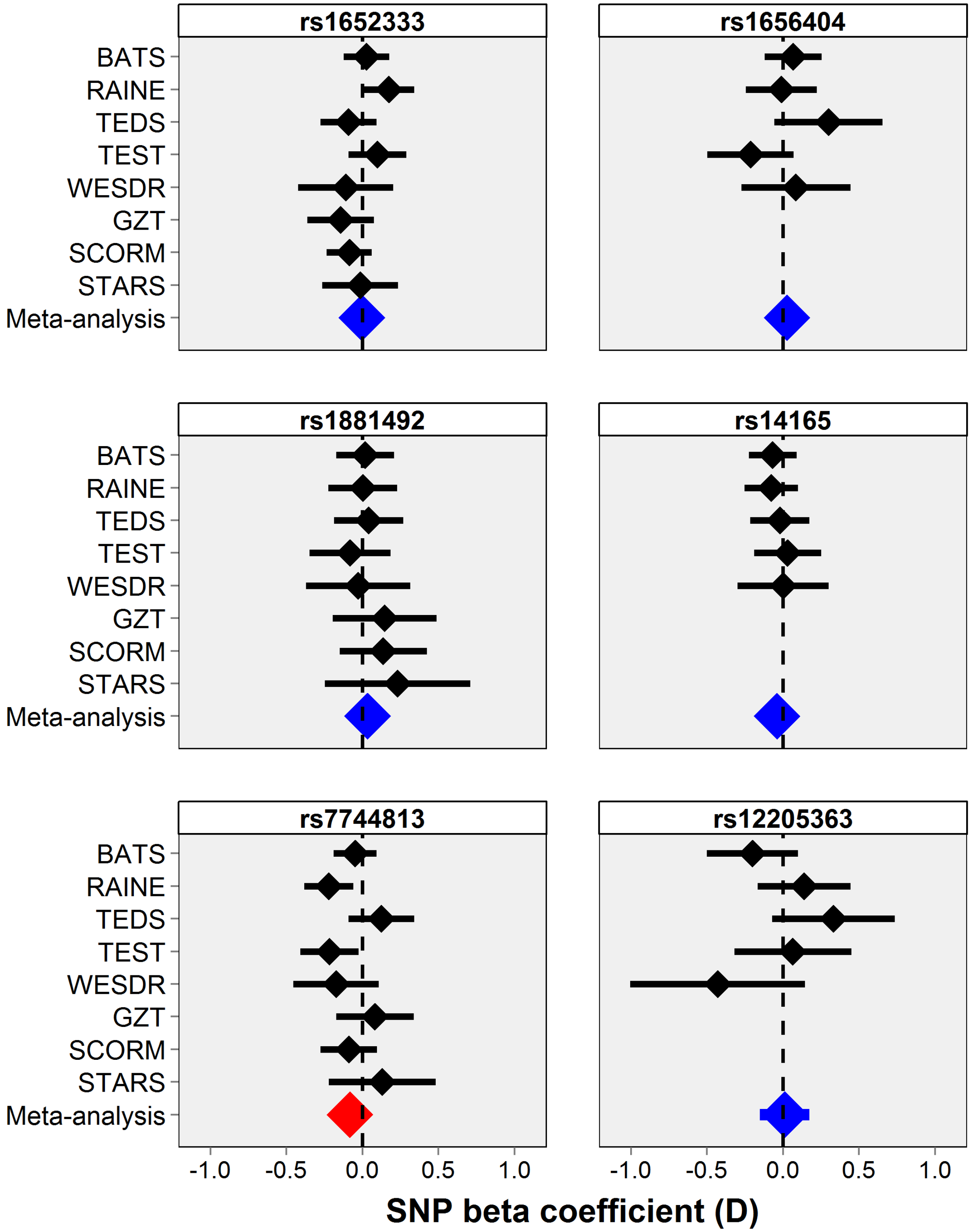

Figure S1 continued:

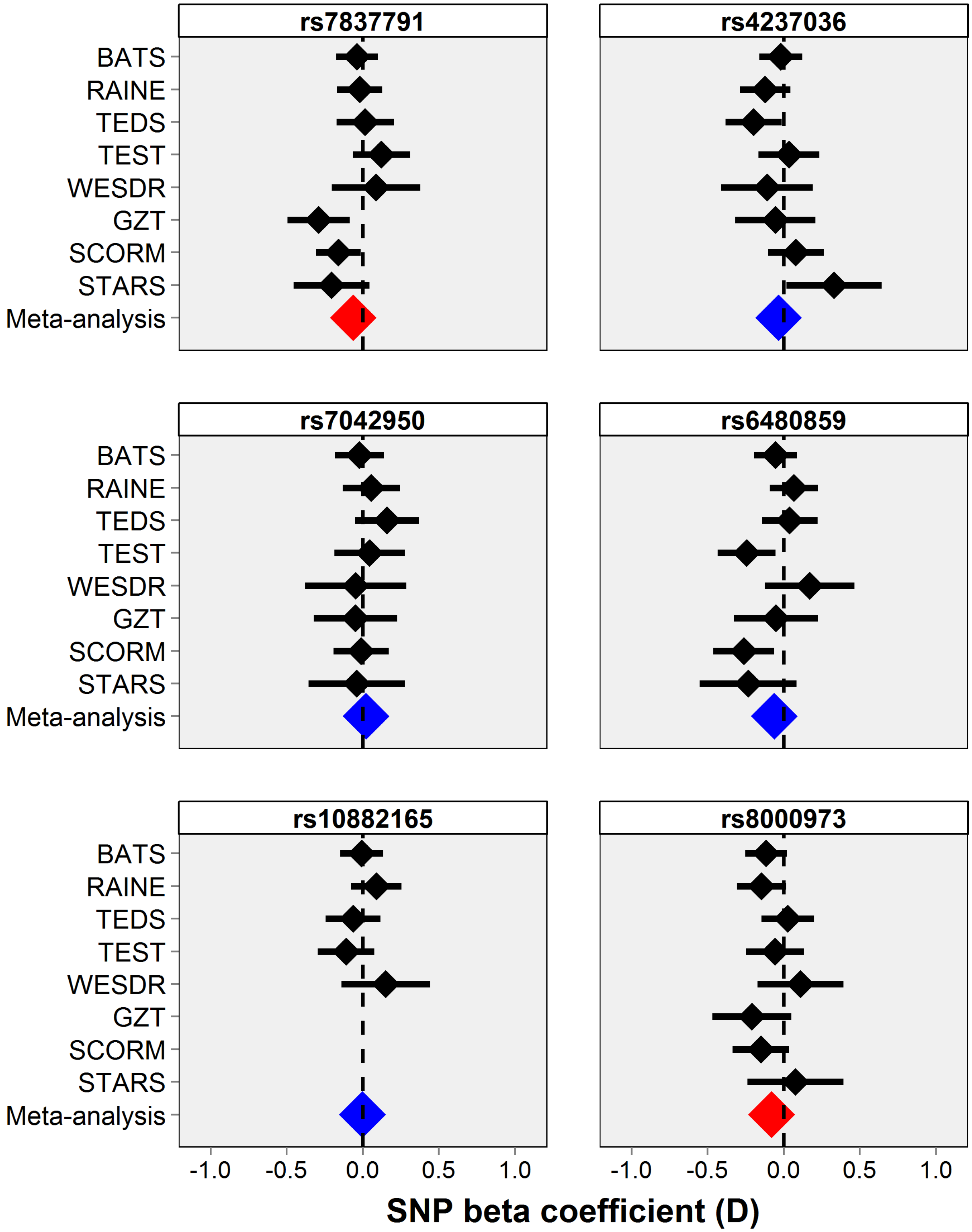

Figure S1 continued:

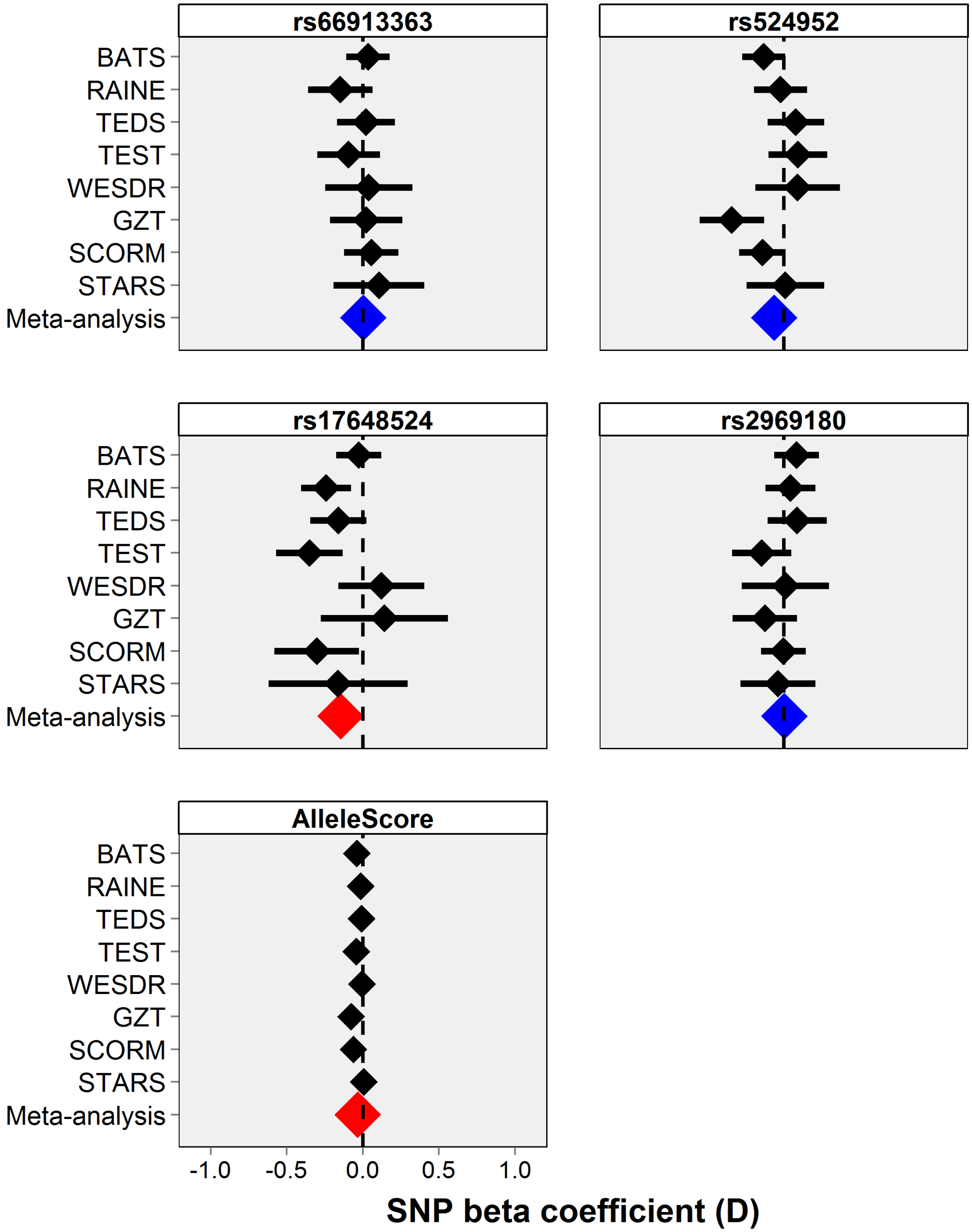

Figure S2. SNP effects in European and Asian meta-analysis samples. Beta coefficients from regression analysis (Dioptres per copy of the risk allele) for association with refractive error in meta-analyses of European and Asian individuals. Panel A: Results for the genetic risk score (black filled symbol) and 12 SNPs associated with refractive error in the ALSPAC longitudinal cohort (the set of "replication SNPs"). Panel B: All 31 SNPs with MAF>0.05 in both Asians and Europeans, plus the genetic risk score (black filled symbol).

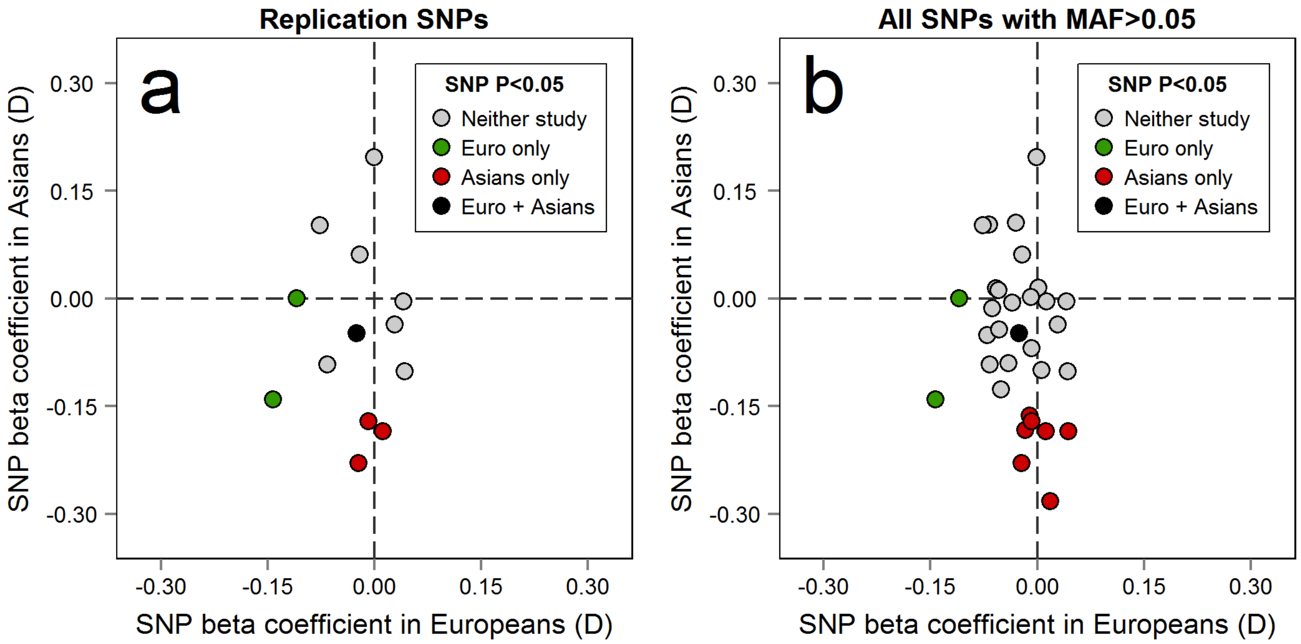

Of the 12 SNPs with MAF>0.05 tested for replication in both ancestry groups, 9 had larger effects in Asians (P=0.07). Of 31 SNPs which had a MAF>0.05 in both ancestry groups, 20 had larger effects in Asians (P=0.07). The effect size of the 31 SNPs available for comparison was approximately 50% larger, on average, in Asian participants than in Europeans (-0.053 D, 95% C.I. -0.015 to -0.092 per copy of the risk allele in Asians versus -0.026 D, 95% C.I. -0.011 to -0.042 per copy of the risk allele in European participants) however this difference was within the range expected to occur by chance (P=0.21).

Figure S3. SNP x nearwork interactions at ages 7 and 15 in the ALSPAC discovery cohort. Refractive error at age 7.5 and age 15 was plotted for ALSPAC participants who were refracted at both ages (N=3,201) after grouping participants by SNP genotype and nearwork exposure. Graphs are presented for the 4 SNPs that showed 3-way SNP x nearwork x age-from-baseline interactions in the LMM analyses. Error bars show 95% CI.

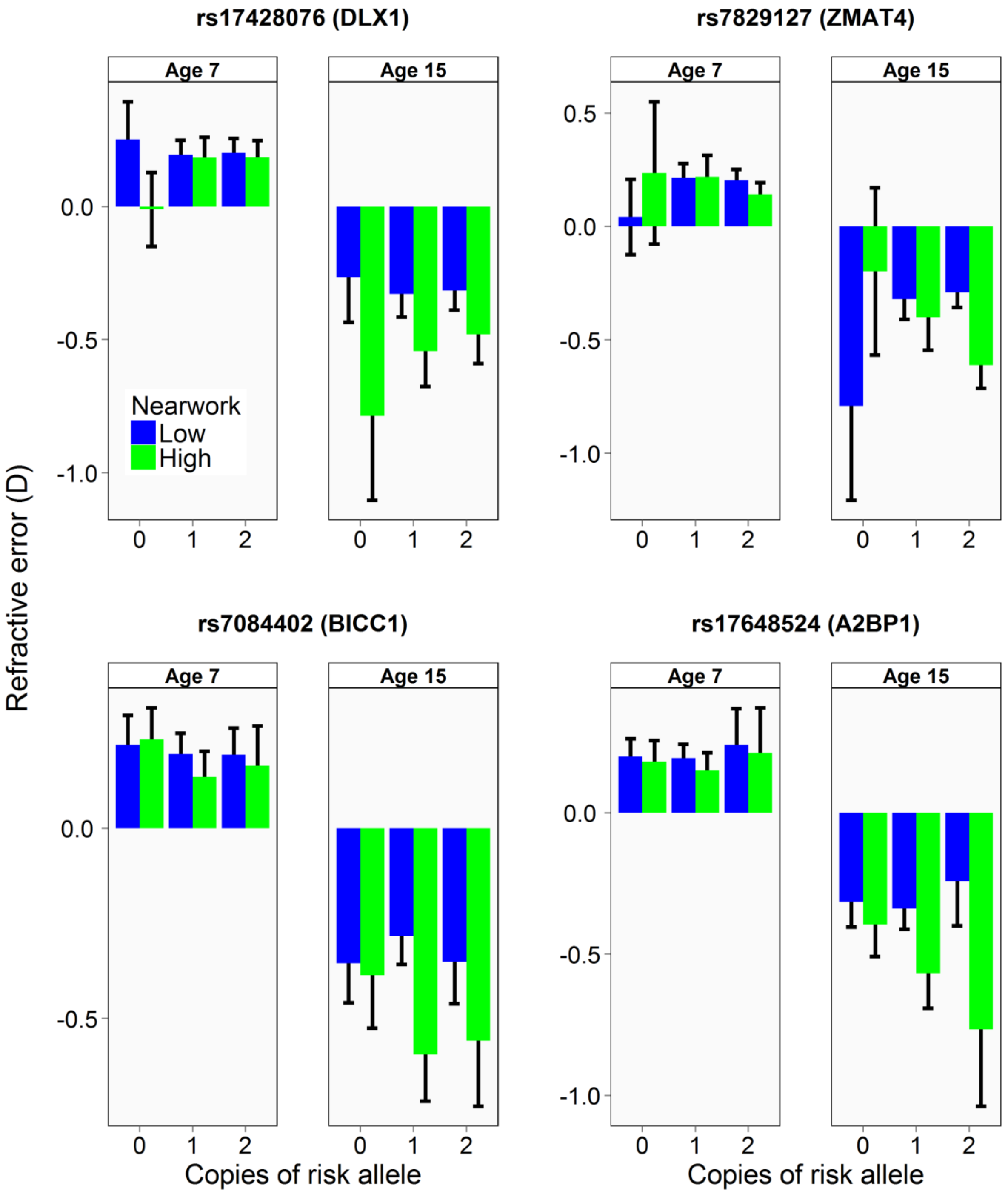

Recruitment of participants and phenotypic assessment

ALSPAC. Ethical approval for the study was obtained from the ALSPAC Ethics and Law Committee and the Local Research Ethics Committees. Participant recruitment has been described previously [5]. Details of the phenotypes available and data access can be found at: http://www.bris.ac.uk/alspac/researchers/data-access/data-dictionary/. Pregnant women with an expected date of delivery between 1st April 1991 and 31st December 1992, resident in the former Avon health authority area in Southwest England, were eligible to participate in this birth cohort study. 13,761 women were recruited. Data collection has been via various methods including self-completion questionnaires sent to the mother, to her partner and after age 5 to the child; direct assessments and interviews in a research clinic. Non-cycloplegic autorefraction (Canon R50 instrument) was performed when participants attended a research clinic visit, at the target ages of approximately 7, 10, 11, 12 and 15 years. DNA samples were available for 11,343 ALSPAC Children, prepared from either blood samples or lymphoblastoid-transformed cell lines. Mothers completed a questionnaire when the children were aged, on average, 8.6 years old. A child was classified as spending a "high" amount of time performing nearwork if their mother reported they spent either "1–2 hours" or "3 or more hours", and as spending a "low" amount of time on nearwork otherwise, in response to the question, "On normal days in school holidays, how much time on average does your child spend each day reading books for pleasure?". The item, "On a weekend day, how much time on average does your child spend each day out of doors in summer?" was used to classify children as spending a "high" amount of time outdoors if the response was "3 or more hours" and as "low" otherwise.

BATS

The Brisbane Adolescent Twin Study is an ongoing study of adolescent and young-adult monozygotic (MZ) and dizygotic (DZ) twin pairs (2720 individuals) and their siblings (1179)[6]. Twins were initially recruited to the study from primary and secondary schools in South East Queensland in 1992, with new twins added at various intervals. In addition, a small number of twins have been recruited through word of mouth, or through the Australian Twin Registry. The study was approved by the human research ethics committee at the QIMR Berghofer Medical Research Institute. Twins have undergone a variety of phenotypic assessments. A 40-ml blood sample is collected from participants and parents at the initial assessment. A subset of participants also completed an extensive eye examination as part of

the Twins Eye Study in Tasmania. Autorefraction was performed using Humphrey-598 Automatic Refractor / Keratometer (Carl Zeiss Meditec, Inc., Miami, Florida, USA).

GZT

The Guangzhou Twin Eye Study was launched in 2006, and it has completed eight consecutive annual follow-up examinations, with more than 1200 twin pairs participating. In brief, twins born in Guangzhou aged 7 to 15 years received annual eye examinations, including cycloplegic refraction, from 2006 onwards. Those with manifest strabismus, amblyopia, nystagmus, post-refractive surgery, or any ocular disease causing best-corrected visual acuity less than 20/25 were excluded from the current analysis. The study was conducted in accordance with the tenets of the World Medical Association's Declaration of Helsinki and was approved by the Ethics Review Board of the Zhongshan Ophthalmic Center of Sun Yat-Sen University. Written informed consent was obtained from the parents or legal guardians of the participants. Cycloplegia was induced with 2 drops of 1% cyclopentolate, administered 5 minutes apart, with a third drop administered after 20 minutes. Cycloplegia and pupil dilation were evaluated after an additional 15 minutes. Cycloplegia was considered complete if the pupil dilated to 6 mm or greater and a light reflex was absent. If not, another 20 minutes observation was taken, and refractive measurement was taken regardless of the presence or absence of light reflex. Refraction was performed with an auto-refractor (Topcon KR-8800, Tokyo, Japan) after cycloplegia. The questionnaire used in the study was designed by a World Health Organization (WHO) working group. It included the questions on indoor and outdoor activities for weekdays and weekend days separately. In each section, daily activity was divided into four types: nearwork activity (including reading, writing, drawing), middle-distance activity (including watching television or movies and playing video games), indoor leisure activity (including singing, housework, dancing in doors), and outdoor activity (including sports, walking outside). Participants were asked to report daily time for each of the activities into 3 categories - not at all, less than one hour or more than one hour. If "more than one hour" was reported, exact time was further specified. During school terms (February to July, September to December) the average time for each type activity was calculated as (5×weekday + 2×weekend)/7. During holidays, the daily visual activity refers to weekend information. In China, every year has 9 months semester days and 3 months summer/winter holidays. The average nearwork and outdoor activity per day in the past year was calculated as (9×semester day time + 3×holidaytime)/12.

RAINE

The Western Australian Birth Cohort (Raine) Study [7] is one of the largest ongoing prospective cohort studies. It was established in 1989 by recruiting 2900 pregnant women at 16-18 weeks of gestation in Perth. The original aim of the study was to investigate how events during pregnancy and at birth influence the health and wellbeing of the newborns. This cohort has gone on to be examined every 2 years by different research groups. At the 20 year follow-up of the Raine Cohort were invited to participate in the Raine Eye Health Study (REHS) and undertake a comprehensive eye examination. This study was approved by the Human Research Ethics Committee at the University of Western Australia. During eye examination, post-cycloplegic autorefraction was performed in 1344 participant using the Nidek ARK-510A (NIDEK Co.Ltd, Japan) autorefractor. As part of the study questionnaire, individuals were asked to report their time spent outdoors and had four possible responses to the question "In the summer, when not working at your job or at school, what part of the day do you spend outside?": none, < ¼ of the day, approximately half of the day and > ¾ of the day. "None" and "<¼ of the day" groups were combined due to low numbers in the "none" category. DNA samples and consents for 1494 participants were available from the previous assessments of the cohort. Individuals with refractive surgery or corneal eye diseases were excluded from the analysis.

SCORM

A total of 1,979 children in grades 1, 2, and 3 from three schools were recruited from 1999 to 2001 with detailed information described elsewhere [8]. The children were examined on the school premises annually by a team of eye care professionals. The GWAS was conducted in a subset of Chinese children of 1,116 participants [9]. The phenotype used in the cross-sectional study was based on the SE measured on the 4th annual examination of the study (children at age 10 to 12 years). Complete post-filtering data on measurements and SNP data were available in 994 SCORM children. Parents were asked through questionnaire to quantify nearwork activity (reading, writing, computer use, playing video games) in hours per day per activity on weekdays and weekends. The average number of outdoor activity hours per day was calculated using the formula: (hours spent on weekday) x 5 + (hours spent on weekend day) x 2)/7. The total outdoor activity was defined as the sum of outdoor leisure and outdoor sporting activities [10].

STARS

STARS is a population-based survey of Chinese families with children residing in the south-western and western region of Singapore. Disproportionate random sampling by 6-month age groups resulted in the recruitment and subsequent eye examination of 3,009 Chinese children between May 2006 and November 2008. Details of the study design and methodology have been previously described [11]. A total of 1,451 samples from 440 nuclear families underwent eye examinations and were included for genome-wide genotyping. In all, 407 children with SE measurement had complete post-filtered genotype data. Near work activities were recorded in number of hours per day. Activities included reading, colouring and drawing, watching television, playing television games, playing hand-held video games and using computers [12]. The outdoor activity questionnaire was similar to that used for SCORM [10].

TEDS

In the initial Twins Early Development Study (TEDS) over 15,000 families of twins born in England and Wales in 1994, 1995 and 1996 were recruited, and the sample remains representative of the UK population [13]. Ethical approval for TEDS and TEDS myopia study has been provided by the Institute of Psychiatry ethics committee, reference number 05/Q0706/228 and PNM/11/12-140 respectively. A subset of 2625 families were selected for the TEDS Myopia study. This sample was selected to include families from TEDS cohort 2 where twins had returned the web questionnaire that included eyesight questions and additional families were added from other cohorts if twins had GWAS data. We excluded from the analyses children with severe current medical problems and families who were not contactable or who lived overseas. Postal questionnaires were sent to a subset of 2,625 families (parents and twins) inviting participation in the myopia study and consent was requested from the parents as well from the twins to contact their optician for a recent refraction. Study questionnaires were sent to the optometrist of 2,283 consenting twins; non-cycloplegic subjective refraction measurements were obtained for 1,996 individuals. DNA samples were available for 3,152 TEDS participants. Multiple child and parent questionnaires, in addition to teacher questionnaires, web-based testing and assessments at home, have been conducted over the twins' life-course; at the age of fourteen a questionnaire was sent to the twins where they asked how much time they spent on a number of extra-curricular activities. The number of hours per week spent on a number of activities, including computer games, reading for fun and hanging outside with friends, was requested.

TEST

Commencing in the late 2000, 1372 participants were recruited to the Twins Eye Study Tasmania through various methods including piggy-backing existing studies where twins had been recruited, utilizing the national twin registry, word-of-mouth and local media publicity and directly approaching schools [14]. Ethical approval was obtained from the Royal Victorian Eye and Ear Hospital, the University of Tasmania, the Australian Twin Registry (ATR). As part of the eye examination, post-cycloplegic autorefraction was completed in all participants using Humphrey-598 Automatic Refractor / Keratometer (Carl Zeiss Meditec, Inc., Miami, Florida, USA). In children, buccal swabs or Oragene saliva samples were collected. In adolescents, or when repeat examination was conducted several years later, a blood sample was taken and those participants who were now adults signed their own consent.

WESDR

The Wisconsin Epidemiologic Study of Diabetic Retinopathy (WESDR) is a population-based observational cohort study of diabetic patients from an eleven-county area in southern Wisconsin since 1979. Participants have gone through an initial and 6 follow-up examinations performed in a van near their residences. Each examination had an extensive ophthalmologic component including measurement of subjective refraction and best corrected visual acuity. For the current analysis, subjective refraction measured at the first visit in adult patients with Type 1 diabetes was used. Further details about recruitment and ophthalmologic exam could be found elsewhere [15].

Supplementary References

1 Cheng, C.-Y. *et al.* Nine loci for ocular axial length identified through genome-wide association studies, including shared loci with refractive error. *Am. J. Hum. Genet.* **93**, 264-277, doi:10.1016/j.ajhg.2013.06.016 (2013).

2 Yazar, S. *et al.* Genetic and Environmental Factors in Conjunctival UV Autofluorescence. *JAMA Ophthalmol.* **133**, 406-412, doi:doi:10.1001/jamaophthalmol.2014.5627 (2015).

3 Davis, O. S. P. *et al.* The correlation between reading and mathematics ability at age twelve has a substantial genetic component. *Nat. Commun.* **5**, 4204, doi:10.1038/ncomms5204 (2014).

4 Hosseini, S. M. *et al.* The association of previously reported polymorphisms for microvascular complications in a meta-analysis of diabetic retinopathy. *Hum. Genet.* **134**, 247-257, doi:10.1007/s00439-014-1517-2 (2015).

5 Boyd, A. *et al.* Cohort Profile: The 'Children of the 90s'--the index offspring of the Avon Longitudinal Study of Parents and Children. *Int. J. Epidemiol.* **42**, 111-127 (2013).

6 Wright, M. J. & Martin, N. G. Brisbane Adolescent Twin Study: Outline of study methods and research projects. *Austral. J. Psychol.* **56**, 65-78, doi:10.1080/00049530410001734865 (2004).

7 Yazar, S. *et al.* Raine Eye Health Study: Design, methodology and baseline prevalence of ophthalmic disease in a birth-cohort study of young adults. *Ophthalmic Genet.* **34**, 199-208, doi:10.3109/13816810.2012.755632 (2013).

8 Saw, S. M. *et al.* A cohort study of incident myopia in Singaporean children. *Invest. Ophthalmol. Vis. Sci.* **47**, 1839-1844, doi:10.1167/iovs.05-1081 (2006).

9 Li, Y. J. *et al.* Genome-wide association studies reveal genetic variants in CTNND2 for high myopia in Singapore Chinese. *Ophthalmol.* **118**, 368–375 (2011).

10 Dirani, M. *et al.* Outdoor Activity and Myopia in Singapore Teenage Children. *Br. J. Ophthalmol.* **93**, 997-1000 (2009).

11 Dirani, M. *et al.* Prevalence of Refractive Error in Singaporean Chinese Children: The Strabismus, Amblyopia, and Refractive Error in Young Singaporean Children (STARS) Study. *Invest. Ophthalmol. Vis. Sci.* **51**, 1348-1355, doi:10.1167/iovs.09-3587 (2010).

12 Low, W. *et al.* Family history, near work, outdoor activity, and myopia in Singapore Chinese preschool children. *Br. J. Ophthalmol.* **94**, 1012-1016 (2010).

13 Haworth, C. M. A., Davis, O. S. P. & Plomin, R. Twins Early Development Study (TEDS): A Genetically Sensitive Investigation of Cognitive and Behavioral Development From Childhood to Young Adulthood. *Twin Res. Hum. Genet.* **16**, 117-125 (2013).

14 Mackey, D. A. *et al.* Twins Eye Study in Tasmania (TEST): Rationale and methodology to recruit and examine twins. *Twin Res. Hum. Genet.* **12**, 441-454 (2009).

15 Klein, B. E., Lee, K. E. & Klein, R. Refraction in adults with diabetes. *Arch. Ophthalmol.* **129**, 56-62 (2011).
